# Methylation of mycovirus DNA is mediated by the RNAi machinery in vegetative hyphae of *Fusarium graminearum*

**DOI:** 10.1093/nar/gkaf478

**Published:** 2025-06-16

**Authors:** Yanfei Wang, Wei Chen, Lihang Zhang, Shuangchao Wang, Jin-Rong Xu, Lihua Guo

**Affiliations:** State Key Laboratory for Biology of Plant Diseases and Insect Pests, Institute of Plant Protection, Chinese Academy of Agricultural Sciences, Beijing 100193, China; School of Life Science, Shanxi Normal University, Taiyuan 030031, China; State Key Laboratory for Biology of Plant Diseases and Insect Pests, Institute of Plant Protection, Chinese Academy of Agricultural Sciences, Beijing 100193, China; State Key Laboratory for Biology of Plant Diseases and Insect Pests, Institute of Plant Protection, Chinese Academy of Agricultural Sciences, Beijing 100193, China; Department of Botany and Plant Pathology, Purdue University, West Lafayette, IN 47907, United States; State Key Laboratory for Biology of Plant Diseases and Insect Pests, Institute of Plant Protection, Chinese Academy of Agricultural Sciences, Beijing 100193, China

## Abstract

DNA cytosine methylation is an important epigenetic mechanism for transposon silencing and gene regulation in fungi, particularly during sexual reproduction. However, its occurrence in vegetative hyphae and role in defense against mycoviruses is unclear. In this study, we demonstrated that genomic-wide cytosine methylation of the tripartite genomovirus FgGMTV1 occurs in the hyphae of *Fusarium graminearum*, a destructive pathogen of wheat and barley worldwide. Elevated methylation levels were predominantly observed in the promoter regions of FgGMTV1, with the highest level reaching 55.87% in the DNA-C fragment MeC5. Methylation of the Rep promoter in DNA-A was showed to be mediated by DNA methyltransferase DIM2 and lead to its transcriptional activity suppression, resulting in a significant reduction in virus accumulation. Furthermore, we uncovered that small RNAs (sRNAs) derived from FgGMTV1 direct the methylation of viral DNA and integrated foreign promoters, which requires the core components of the RNAi machinery, including the Ago and Dicer genes. Deletion of *dcl1/2* or *ago1/2* in FgGMTV1-infected strains resulted in an increase in virus accumulation and defects in hyphal growth, stress response, and plant infection. Taken together, our findings reveal that RNAi-mediated DNA methylation occurs in vegetative hyphae and plays a crucial role in antiviral defense mechanisms in fungi.

## Graphical Abstract

**Graphical Abstract:**
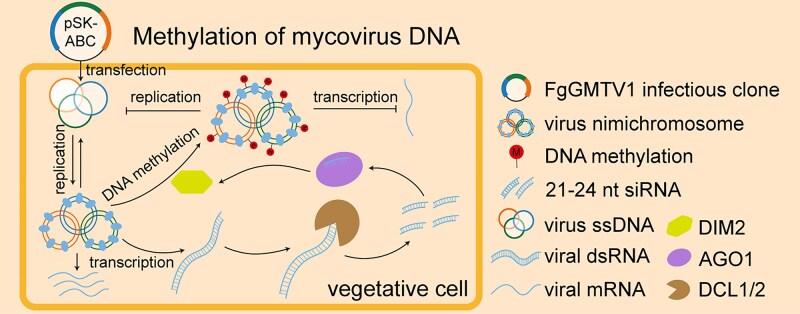

## Introduction

DNA cytosine methylation is a highly conserved epigenetic modification that plays pivotal roles in defending against nucleic acid invasions, maintaining genome integrity, and regulating gene expression in eukaryotes [1–3]. This modification involves the enzymatic conversion of cytosine bases to 5-methylcytosine by DNA methyltransferases (DNMTs). DNMTs are broadly categorized into two functional classes: “*maintenance*” methylation, which preserves methylation patterns post-DNA replication at hemimethylated sites, and “*de novo*” methylation, which introduces methylation at previously unmethylated cytosines [2]. In fungi, DNA methylation is predominantly associated with two genome defense mechanisms: repeat-induced point mutation (RIP) and methylation-induced premeiotically (MIP), both of which function during sexual reproduction [4, 5]. In the model filamentous ascomycete *Neurospora crassa*, DNA methylation occurs in transposons that have been rendered A:T-rich by the RIP defense system, a process facilitated by deficient in methylation-2 (DIM-2) and accompanied by histone H3K9 methylation [6–8]. Similarly, in *Ascobolus immersus*, DNA methylation specifically targets duplicated DNA sequences during MIP, with DNMT Masc1 acting as a key mediator prior to meiosis [9, 10]. Although DNMTs and genome-wide DNA methylation patterns have been characterized across diverse fungal species, these activities are predominantly observed in repetitive sequences during sexual reproduction [11, 12]. Comparatively, little is known about the occurrence and role of these methylation events during the vegetative cycle of fungi.

In plants and animals, viral DNA methylation serves as a critical defense mechanism against viral infections [13, 14]. For instance, when geminiviruses infect plants, their genomic DNA undergoes varying degrees of methylation [15]. The resistance genes *Ty-1*/*Ty-3* in tomato, which encode the RNA-dependent polymerase RDRγ, confer resistance to tomato yellow leaf curl virus (TYLCV) through DNA methylation [16]. Transgenic plants expressing the *β*-Glucuronidase (GUS) reporter gene under the control of the tomato leaf curl virus (TLCV) promoter exhibit GUS silencing upon TLCV infection due to extensive promoter methylation [17]. These observations underscore the broad-spectrum defensive capabilities of DNA methylation in plants against geminiviruses. In animals, methylation of DNA tumor viruses serves to regulate the viral life cycle by influencing the transcription and expression of viral genes [18]. To circumvent this defensive, viruses have evolved suppressor proteins that disrupt DNA methylation or interfere with the biosynthesis of S-adenosyl-L-methionine (SAM), a crucial molecule in the DNA methylation pathway [15]. Despite its significance in other systems, DNA methylation of fungal viruses remains unexplored, likely due to the rarity of DNA mycoviruses in nature [19].

RNA interference (RNAi) is a conserved gene regulatory mechanism in eukaryotes including plants, animals, and fungi. It mediates post-transcriptional gene silencing (PTGS) by degrading or repressing target mRNAs and transcriptional gene silencing (TGS) by directing epigenetic modifications to the genome [20–22]. In plants, RNA-directed DNA methylation (RdDM) is the primary pathway for establishing *de novo* DNA methylation, relying on core RNAi machinery proteins such as Dicer-like (DCL) and Argonaute (AGO) [23]. In animals, PIWI-interacting RNAs (piRNAs) drive *de novo* methylation of differentially methylated regions (DMRs), independently of the RNAi pathway [24–26]. In *Schizosaccharomyces pombe*, small RNAs (sRNAs) induce TGS and co-transcriptional gene silencing (CTGS) through H3K9 methylation rather than DNA methylation [27]. Notably, *Freitag*  *et al.* demonstrated that the RNAi pathway does not participate in the regulation of DNA methylation in *N. crassa* [28]. However, recent studies have revealed that DNA methylation in the human-pathogenic basidiomycete *Cryptococcus neoformans* is mediated by DNMT5, which contains sucrose nonfermenting (SNF2_N) and helicase_C domains homologous to those required for RdDM in plants [29–32]. Additionally, hypermethylation of transposable element (TE)-related sRNA clusters has been observed in basidiomycetous *Pleurotus* fungi [33]. Although these findings suggest the presence of an RdDM-like mechanism in the fungal kingdom, no direct evidence has been reported to date.

Fusarium graminearum gemytripvirus 1 (FgGMTV1), a member of the *Genomoviridae* family, is a tripartite circular single-stranded DNA mycovirus isolated from *F. graminearum*, an important pathogen causing Fusarium head blight (FHB) in cereal crops such as wheat, leading to significant yield loss and mycotoxin contamination [34, 35]. The first two genomic segments, DNA-A and DNA-B, encode a replication initiation protein (Rep) and a capsid protein (CP), respectively, while DNA-C encodes three proteins: p26, p9 (both with unknown functions), and p18, which facilitates asymptomatic viral infection in *F. graminearum* [36, 37]. The replication process of FgGMTV1, which is speculated to resemble that of geminiviruses, occurs in the nucleus via a rolling-circle mechanism, generating double-stranded DNA (dsDNA) intermediates that bind to host histones and form minichromosomes [38]. Small RNA sequencing has revealed that FgGMTV1-infected *F. graminearum* strains produce abundant viral siRNAs, suggesting activation of host RNA silencing mechanisms [39]. Moreover, a FgGMTV1-based virus-induced gene silencing (VIGS) system has been developed to attenuate hypervirulent *F. graminearum* strains, offering a potential strategy for FHB control [40].

In this study, we demonstrate that *F. graminearum* employs viral DNA methylation as a defense mechanism against FgGMTV1. Methylation of the Rep promoter in DNA-A, mediated by the DNMT DIM2, suppresses Rep transcriptional activity, thereby inhibiting virus accumulation. we further reveal a novel interplay between viral-derived sRNAs and the core RNAi proteins DCL1, DCL2, and AGO1, which collectively guide the methylation of both viral DNA and integrated foreign promoters. Notably, although DCL1 and AGO2 primarily mediate the sex-specific RNAi pathway in *F. graminearum* [41], our findings highlight functional redundancy within the DCL and AGO families, with DCL2 and AGO1 playing pivotal roles in the antiviral pathway. This study provides groundbreaking evidence that DNA methylation in fungi is dependent on RNAi, marking a significant advancement in fungal epigenetics. Furthermore, our results underscore the critical role of DNA methylation in fungal antiviral defense, shedding light on the complex dynamics of host-pathogen interactions.

## Materials and methods

### Fungal strains and culture conditions

The virus-free wild-type strain PH-1 of *F. graminearum* was used as the parent strain [42]. All fungal strains used in this study are listed in Supplementary Table S1. The strains were cultured in the potato dextrose agar (PDA) medium for 3–5 days at 25°C for morphological observation or in the cellophane-covered PDA medium for DNA and RNA extraction. For spore counting, the strains were cultured in the carboxymethyl cellulose (CMC) liquid medium for 5–7 days at 25°C to obtain the spores. For sexual reproduction experiments, 7-day-old aerial hyphae grown on carrot agar cultures (250 g/L) were pressed down using sterile 0.1% Tween-20 and subsequently cultured under black light at 25°C. The formation and development of perithecia, asci, ascospores, and ascospore discharge were examined as previously described [43, 44]. To count the number of ascospore discharges, the carrot agar was inverted for 12 h to allow mature perithecia to release ascospores onto the petri dish covers. The ascospores were then washed off the slide using 1 mL of sterile water, and their number was enumerated.

### Strain and plasmid construction

Mutant alleles for targeted gene deletion were generated using double-joint (DJ) PCR, as previously described [45]. Briefly, 5′ and 3′ flanking regions of each gene were amplified using the primer pairs listed in Supplementary Table S1, and the amplified sequences were fused with the hygromycin resistance gene (*HPH*) cassette driven by the constitutive trpC promoter. Then PEG-mediated protoplast transformation of *F. graminearum* was performed, as previously described [40]. All putative gene-deletion mutants were identified via PCR using the relevant primers (Supplementary Table S1). *F. graminearum dcl1*, *dcl2*, and *dcl1/2* mutants [46] were kindly provided Jin-Rong Xu, Purdue University, West Lafayette, Indiana, USA. To complement the *dim2* deletion mutant (*dim2*), a fusion construct containing *dim2* with its own promoter, neomycin resistance gene (*NEO*), and 3′-flanking region of *dim2* was generated via DJ PCR. The fusion construct was further transformed into *dim2* to generate the complementary strain, *dim2*/*DIM2*. The transformants were selected using geneticin (G418).

To generate the constructs to analyze the Rep promoter activity, 500-nt sequence upstream of the DNA-A-encoded Rep ATG was PCR-amplified and cloned between the *Kpn* I and *Bam*H I sites of pGTN (RP27-GFP) to replace the RP27 promoter of GFP gene and generate pRep(500)-GFP using Seamless Cloning Kit (LABLEAD lnc., Beijing, China). The empty pGTN vector, RP27-GFP, or the pRep(500)-GFP vector were transformed into PH-1 and *dim2* to generate PH-1::RP27-GFP, *dim2*::RP27-GFP, PH-1::pRep(500)-GFP, and *dim2*::pRep(500)-GFP.

The transfection of *F. graminearum* with the viral infectious clone pSK-ABC was performed using a PEG-mediated protoplast transfection method, as previously described [40].

To verify the effect of small RNA-mediated DNA methylation on RP27 promoter activity, we transformed the empty pGTN vector with RP27-GFP into PH-1, *dcl1*, *dcl2*, *ago1*, and *ago2* strains to generate PH-1::RP27-GFP, *dcl1*::RP27-GFP, *dcl2*::RP27-GFP, *ago1*::RP27-GFP, and *ago2*::RP27-GFP strains, respectively. To construct VIGS vectors for the RP27 promoter, 150 bp sequences of RP27 promoter were cloned from the pGTN vector and inserted into the FgGMTV1 VIGS vector p26-D4 digested with *Nsi* I and *Age* I [35]. The 150-bp sequence of GUS gene was also cloned from the pCAMBIA1301 vector and inserted into the p26-D4 vector. Plasmids p26-D4-GUS, p26-D4-RP27F (sense), and p26-D4-RP27R (antisense) were also constructed. All primers used here are listed in Supplementary Table S2. The established VIGS vectors were transfected into PH-1::RP27-GFP, *dcl1*::RP27-GFP, *dcl2*::RP27-GFP, *ago1*::RP27-GFP, and *ago2*::RP27-GFP strains to obtain corresponding strains. Protoplast preparation and viral transfection were performed as previously described [35].

### RNA extraction and RT-qPCR

RNA was extracted using the FastPure Universal Plant Total RNA Isolation Kit (Nanjing Vazyme Biotech, Nanjing, China) and reverse-transcribed into cDNA synthesis was carried out using Hifair III 1st Strand cDNA Synthesis Kit (Yeasen Biotechnolog Co. Ltd., Shanghai, China). Real-time qRT-PCR was performed using QuantStudio 5 (ABI) with Hieff qPCR SYBR Green MasterMix (Yeasen Biotechnology Co. Ltd., Shanghai, China). *FgEF-1α* was used as the internal control. All primer sequences are listed in Supplementary Table S2.

### DNA extraction, southern blotting analysis, and qPCR

Total DNA was extracted from fungi using the cetyltrimethylammonium bromide method. Southern blotting was used to analyze viral DNA accumulation. Briefly, 1 μg of total DNA was separated on 1.5% agarose gels. After denaturation and neutralization of the agarose gels, total DNA was transferred to Hybond N + nylon membranes (Cytiva, Washington, USA). Hybridization and signal detection were performed using the DIG-High Prime DNA Labeling and Detection Starter Kit II (Roche, Basel, Switzerland). Three DNA probes, DNA-A, DNA-B, and DNA-C, were used to detect FgGMTV1, as previously described [36]. To detect viral DNA accumulation using qPCR, partial Rep fragments of 183 bp, partial CP fragments of 181 bp, and partial p26 fragments of 200 bp were amplified. All primer sequences used for qPCR are listed in Supplementary Table S2.

### Responses of mutant strains to various stressors and pathogenicity assays

To determinate the responses of mutants to various stressors, mycelial plugs (5 mm in diameter) taken from the periphery of the 4-day-old colony of each strain were inoculated into the PDA medium mixed with hydrogen peroxide (0.1% (v/v) H_2_O_2_) and cell membrane-disrupting agents (0.05% (w/v) SDS), respectively. After the plates were incubated at 25°C for 4 days, colony diameter in each plate was measured. Each experiment was repeated three times. To assess the pathogenicity in the flowering wheat heads (cultivar Jimai22), a small equisized mycelial plug was inoculated in the glume of a spikelet and cultured in the field for 15 days. To detect virulence on seedling leaves (cultivar Jimai22), a 5-mm mycelial plug of each strain was inoculated into the center of the leave, and cultured at 25°C and 100% humidity with 12 h of daylight, and examined at 7 days. Each experiment was repeated three times.

### Evaluation of GFP fluorescence

Sterile glass coverslips (20 × 20 mm) were placed on cellophane overlaying the PDA medium, and a small mycelial plug was inserted at the edge of the coverslip. When the mycelia grew on the coverslip, the coverslip with the attached mycelia was transferred to a glass slide, and GFP fluorescence was observed using a microscope (Carl Zeiss, Oberkochen, Germany). GFP fluorescence of spores, which were obtained by culturing the strains in the CMC liquid medium for 5–7 days at 25°C, was observed using a microscope (Carl Zeiss, Oberkochen, Germany). GFP fluorescence intensity was quantified using the ImageJ software [47].

### DNA bisulfite sequencing analysis

Total DNA (500 ng) was subjected to bisulfite treatment using the DNA Bisulfite Transformation Kit (TIANGEN biotech CO. LTD., Beijing, China), according to the manufacturer's instructions. Bisulfite-treated DNA was used as the template for PCR amplification. The PCR products were ligated into the pMD18-T vector. Approximately 30 colonies were sequenced, and cytosine methylation was analyzed using the CyMATE program (http://cymate.org/cymate.html) [48]. Sequences with the same cytosine methylation status were counted as clones. At least three technical replicates of bisulfite treatment were performed for each viral infection experiment.

### Virus-derived small RNA analysis

Mycelial masses were collected at the same time point after a 4-day culture period. Total RNA was extracted using the RNA Extraction Kit (TIANGEN Biotech CO. LTD., Beijing, China). RNA quality was evaluated with a BioAnalyzer 2100 system (Agilent Technologies, Santa Clara, CA, USA). The cDNA libraries of sRNA were constructed with the QIAseq sRNA Library Kit (Qiagen, Hilden, Germany). In brief, 3′ and 5′ adapters were sequentially ligated to sRNAs, followed by unique molecular identifier (UMI)-based first-strand cDNA synthesis. Subsequent steps included cDNA purification and PCR amplification using a universal forward primer combined with index-containing reverse primers. Library quality control was performed using the 2100 Bioanalyzer (Agilent Technologies, Santa Clara, CA, USA). Sequencing was conducted on an Illumina NextSeq 6000 platform (Illumina, San Diego, CA, USA). Raw sequencing data were processed by removing low-quality reads and adapter contaminants using FASTX-Toolkit (v0.0.13) and filtering reads outside the 21–24 nt size range using Cutadapt (v4.1). The remaining reads with lengths of 21–24 nt were mapped to the genome of FgGMTV1 (GenBank accession: GCA_018594965.1) using the Bowtie software (http://bowtie-bio.sourceforge.net). The number of vsiRNA reads was normalized to reads per million (RPM) values (RPM = number of reads on a sRNA × 10^6^/total number of reads) according to the number of total reads in the corresponding small RNA library. The library construction and sequencing were performed at Shanghai Biotechnology Corporation.

### Statistical analysis

Data are represented as the mean ± standard deviation (SD). Statistical analysis was performed using GraphPad Prism version 8.0. Significant differences between the control and treatment groups were analyzed using the one-way analysis of variance (ANOVA) followed by Dunnett's post-hoc test for pairwise comparisons. Statistical significance was set at *P* < 0.05 (*P < 0.05, **P < 0.01, and ***P < 0.001). The sample size (n) for each statistical analysis were reported in the corresponding “figure legends.”

## Results

### FgGMTV1 genome is a DNA methylation target

**Figure 1.**
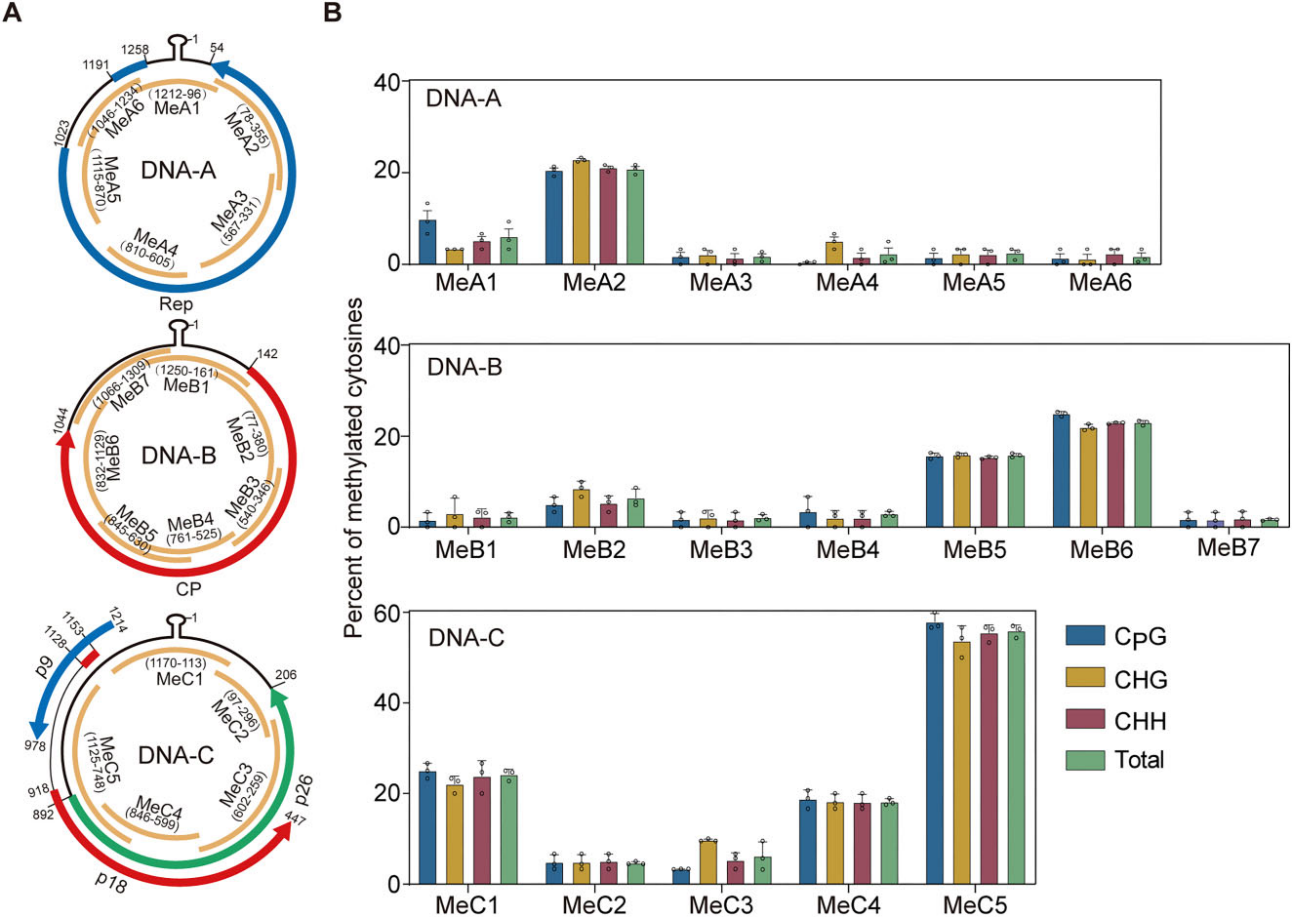
The genome-wide DNA methylation of FgGMTV1. (**A**) Three components of FgGMTV1 were depicted in circular forms, with six fragments of DNA-A (MeA1–6), seven fragments of DNA-B (MeB1–7) and five fragments of DNA-C (MeC1–5) used for bisulfite sequencing analysis indicated as orange lines. The blue arrow in DNA-A represents the open reading frame (ORF) of Rep. The red arrow in DNA-B represents the ORF of CP. The blue, red, and green arrows in DNA-C represent the ORFs of p9, p18, and p26, respectively. (**B**) Percentage of methylated cytosines in each fragment of FgGMTV1 determined by bisulfite sequencing with DNA isolated 4-day-old PDA cultures. For each fragment, 30 clones were sequenced to estimate the mean and standard deviation. Values are represented as the mean ± standard deviation (SD) (*n* = 3 independent experiments).

Methylation of viral genomes is an important epigenetic defense mechanism in animals and plants [13, 14]. To determine whether the FgGMTV1 genome is targeted by DNA methylation in *F. graminearum*, bisulfite sequencing was performed with the DNA-A, DNA-B, and DNA-C sequences of FgGMTV1 that were divided into six (MeA1–6), seven (MeB1–7), and five (MeC1–5) fragments, respectively (Fig. 1A). For DNA-A, fragment MeA2 had the highest methylation level (20.73%), with majority of methylation occurring at C_P_G, CHG, and CHH (Fig. 1B and Supplementary Fig. S1). The methylation level in other fragments of DNA-A range from 1.67% to 5.98%. For DNA-B, the fragments with the highest to lowest methylation levels were MeB6 (22.93%), MeB5 (15.79%), MeB2 (6.36%), MeB4 (2.86%), MeB1 (2.16%), MeB3 (1.92%), and MeB7 (1.66%). For DNA-C, fragment MeC5 had the average cytosine methylation level of 55.87%, with 57.88% of C_P_G, 53.67% of CHG, and 54.43% of CHH (Fig. 1B and Supplementary Fig. S1).

Through FgGMTV1 transcript mapping analysis, we observed that cytosine methylation patterns in the viral genome displayed preferential enrichment within or proximal to promoter regions. Specifically, three key methylated fragments were identified: MeA2 (78–355 nt, DNA-A), putatively located in the promoter region controlling expression of the DNA-A-encoded Rep protein; MeC5 (748–1125 nt, DNA-C), mapped to the promoter driving expression of the DNA-C-encoded p26 protein; and MeC1 (1170–113 nt, DNA-C), associated with the regulatory sequence governing p9/p18 expression (Fig. 1A) [36, 37]. These results provide direct evidence that FgGMTV1 is affected by cytosine methylation and that methylation is particularly enriched in the sequences within or near promoters, suggesting epigenetic regulation of viral gene expression.

### DNMT DIM2 plays a critical role in the methylation of FgGMTV1

**Figure 2.**
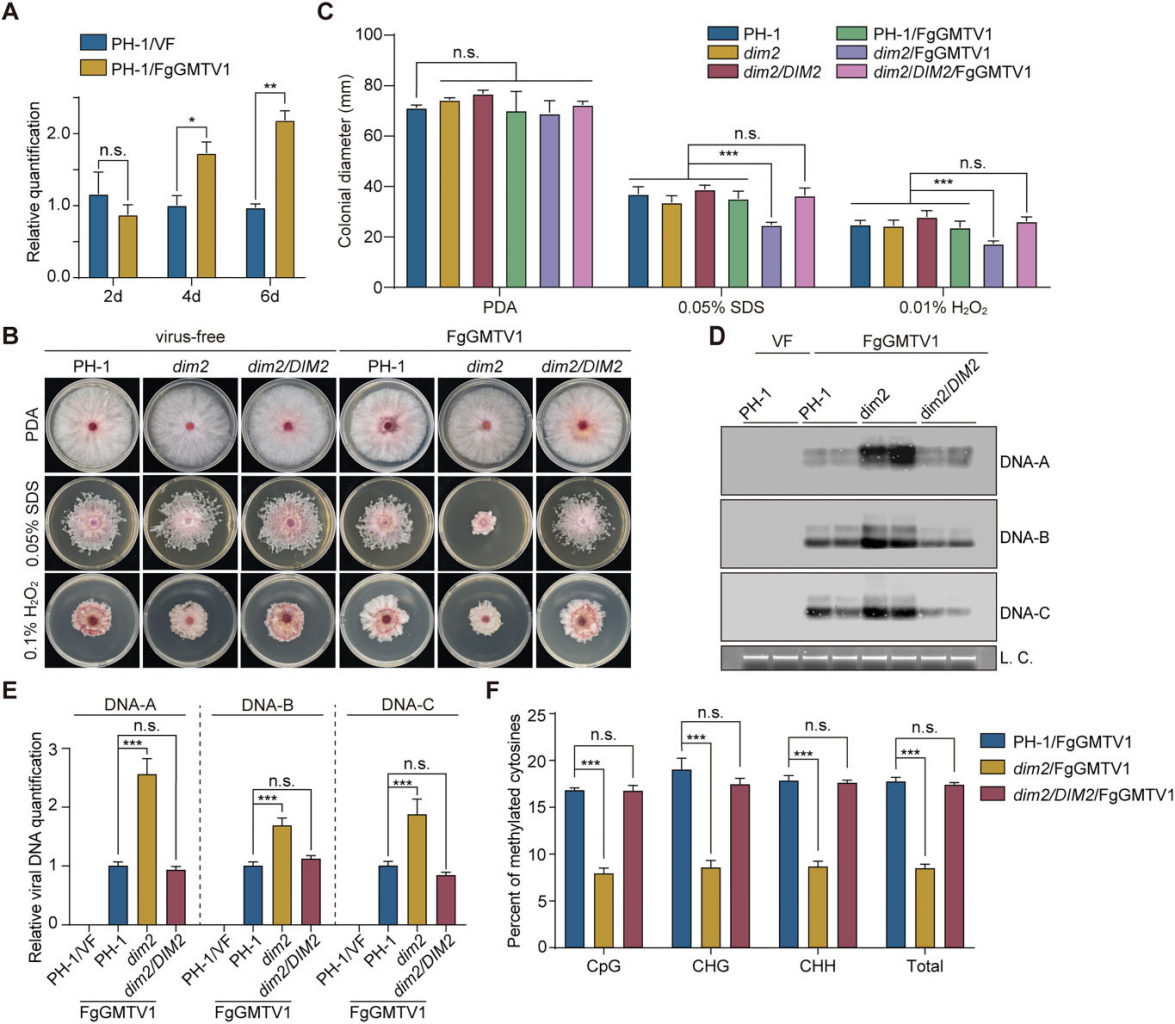
DIM2 plays a critical role in the methylation of FgGMTV1. (**A**) Expression analyses of *dim2* in virus-free and FgGMTV1-infected strains on the PDA medium for 2, 4, and 6 days in the dark by RT-qPCR. The expression level of *FgEF-1α* was used as the internal control. (**B**) Colony morphologies of the virus-free (top) and FgGMTV1-infected (bottom) strains on PDA with or without 0.05% SDS and 0.1% H_2_O_2_ after incubation for 3 or 5 days. (**C**) colony diameters of the marked strains on PDA after incubation for 3 or 5 days. (**D**) Southern blots of genomic DNA isolated from 4-day-old PDA cultures in the dark were hybridized with probes A, B, and C that are specific for DNA-A, DNA-B, and DNA-C of FgGMTV1, respectively. LC, loading control to show equal amounts of genomic DNA in each lane. (**E**) The abundance of DNA-A, DNA-B, and DNA-C of FgGMTV1 in the same samples was assayed by qPCR. The *FgEF-1α* gene was used as the internal control. (**F**) Percentage of methylated cytosines in fragment MeA2 of FgGMTV1 in FgGMTV1-infected PH-1, *dim2*, and *dim2/DIM2* strains determined by bisulfite sequencing. For **A**, **C**, **E**, and **F**, values are represented as the mean ± SD (*n* = 3 independent experiments). Statistical significance was determined using one-way ANOVA followed by Dunnett's post-hoc test. *, *P* < 0.05; **, *P* < 0.01; ***, *P* < 0.001; and n.s., not significant.

In plants, mammals, and fungi, DNA methylation is catalyzed by conserved DNMTs, using SAM as the methyl donor. Through homology searches using DNMTs of *N. crassa*, the following putative DNMT genes were identified in *F. graminearum*: *dim2* (FGSG_10766) and *rid* (FGSG_08648) [49]. The *rid* acts as a pseudogene as it contains a UA^644^G premature stop codon that requires stage-specific editing to encode a full-length functional protein during sexual reproduction [50, 51]. Here, we analyzed the *dim2* levels in both the virus-free and FgGMTV1-infected wild-type strain PH-1 using reverse transcription-quantitative polymerase chain reaction (RT-qPCR). The abundance of *dim2* transcripts was significantly elevated in four-day cultures of PH-1/FgGMTV1 compared to that in the virus free culture (Fig. 2A). This result indicates that FgGMTV1 infection significantly alters the accumulation of the *dim2* transcripts in *F. graminearum*.

To determine the function of DIM2 in FgGMTV1 infection, *dim2* was knocked out using a homologous recombination strategy. Two independent *dim2* deletion mutant strains were obtained, and gene deletion was confirmed via PCR (Supplementary Fig. S2A). We transformed protoplasts of *dim2* mutants with a functional *dim2* gene under the control of its native promoter to produce the *dim2/DIM2* complementation strain (Supplementary Fig. S2B). The infectious clone of FgGMTV1 was then transfected into PH-1, *dim2*, and *dim2/DIM2* strains via polyethylene glycol (PEG)-mediated protoplast transfection. Southern hybridization analysis confirmed that all transfectants were infective and verified the successful establishment of FgGMTV1-infected PH-1, *dim2*, and *dim2/DIM2* (Fig. 2B and D). When quantified by southern blot and qPCR analyses, the abundance of FgGMTV1 was significantly higher in the *dim2* mutant than in PH-1 and the *dim2/DIM2* transformant (Fig. 2D and E). Bisulfite sequencing analyses with fragments MeA2, MeB6, and MeC5 showed that their methylation levels were significantly reduced in the *dim2* mutant in comparison with PH-1 and *dim2/DIM2* (Fig. 2F and Supplementary Fig. S2C–F). These results indicate that DIM2 plays a critical role in the methylation of FgGMTV1 in *F. graminearum*.

To characterize the function of DIM2-mediated FgGMTV1 methylation, we assessed mycelial growth, sexual/asexual reproduction, responses to stress, and virulence. Compared to PH-1 and *dim2/DIM2*, the FgGMTV1-infected *dim2* showed no significant difference in colony morphology, mycelial growth rate, and conidiation (Fig. 2B and C, and Supplementary Fig. S3A). During sexual reproduction, all strains produced normal perithecia with mature asci and exhibited normal ascospore discharge (Supplementary Fig. S3B and C). This is similar to *N. crassa* but distinct from *Cryphonectria parasitica* [52].

The FgGMTV1-infected strains were also examined for changes in response to oxidative (H_2_O_2_) and cell membrane (sodium dodecyl sulfate [SDS]). The FgGMTV1-infected *dim2* displayed an increased sensitivity to SDS and H_2_O_2_ (Fig. 2B and C). In infection assays, the virulence of the strain *dim2*/FgGMTV1 showed similar characteristics to the strain PH-1/FgGMTV1 in flowering wheat heads and wheat seedlings leaves (Supplementary Fig. S3D–F). Our findings further revealed that genome methylation of FgGMTV1 enhances the fungi's adaptability to abiotic stress.

### DIM2-mediated methylation suppresses FgGMTV1 Rep promoter-dependent transcriptional activity

**Figure 3.**
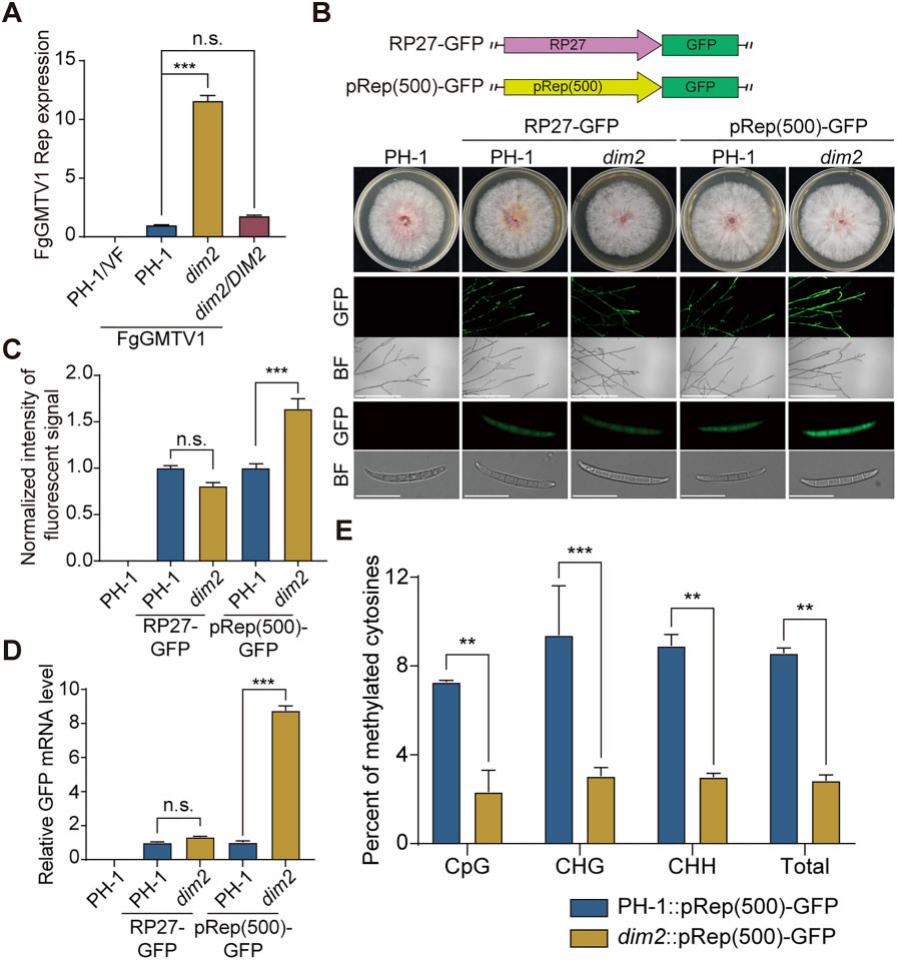
DIM2-mediated methylation of viral genome suppresses promoter-dependent transcriptional activities. (**A**) Analysis of the transcription of FgGMTV1-encoded *Rep* in FgGMTV1-infected PH-1, *dim2*, and *dim2/DIM2* strains by RT-qPCR. (**B**) Schematic diagram of GFP expression driven by the FgGMTV1-encoded Rep promoter (pRep(500)). Colony morphology and green fluorescence intensity of mycelia and spores in PH-1 and *dim2* strains transformed with RP27-GFP and pRep(500)-GFP. All strains were cultured in the PDA medium for mycelia and in the CMC medium for spores. Bar = 0.5 mm (Upper); bar = 25 μm (Lower). (**C**) Normalized intensity of fluorescent signal in PH-1 and *dim2* strains transformed with RP27-GFP and pRep(500)-GFP. The fluorescence intensity was quantified using the ImageJ software, with those in PH-1::RP27-GFP and PH-1::pRep(500)-GFP strains set as 1.00. (**D**) Relative expression levels of GFP in 4-day-old PDA cultures of PH-1 and *dim2* strains expressing RP27-GFP and pRep(500)-GFP assayed by qRT-PCR with *EF-1α* as the internal control. (**E**) Cytosine methylation in the Rep promoter in PH-1 and *dim2* strains transformed with pRep(500)-GFP. Samples were collected as described in (**C**). For **A**, **C**, **D**, and **E**, values are represented as the mean ± SD (n = 3 independent experiments). Statistical significance was determined using one-way ANOVA followed by Dunnett's post-hoc test. **, *P* < 0.01; ***, *P* < 0.001; and n.s., not significant.

The promoter DNA methylation usually inhibits gene transcription in animals, plants, and fungi [3]. In the context of plant geminiviruses, the Rep protein holds a pivotal role in viral genome replication through the rolling circle replication mechanism [15]. Specifically, the MeA2 fragment is situated precisely 500 bp upstream of the DNA-A-encoded Rep open reading frame (ORF) (Rep promoter). When assayed by RT-qPCR, the expression of Rep in the FgGMTV1-infected *dim2* mutant was significantly elevated compared to PH-1 and *dim2/DIM2* (Fig. 3A), indicating a suppressive role of DIM2-mediated methylation. Thus, we hypothesized that DIM2 induces DNA hypermethylation of the Rep promoter, leading to Rep transcriptional inhibition, thereby inhibiting the accumulation of FgGMTV1.

To test this hypothesis, we cloned the 500-bp sequence upstream of the Rep ORF before the green fluorescent protein (GFP) reporter gene (pRep(500)-GFP) and transformed pRep(500)-GFP into the wild-type strain PH-1 (Fig. 3B). The GFP fluorescent signal was observed in all G418-resistant transformants with pRep(500)-GFP, suggesting that the 500-bp sequence upstream of the Rep ORF was functional (Fig. 3B). Then, we transformed the pRep(500)-GFP into the *dim2* mutant strain. The *ribosomal protein 27* (RP27) promoter with a GFP reporter (RP27-GFP) was used as a negative control. The GFP fluorescent signal assay shows that while the intensity of green fluorescence of RP27-GFP exhibited a similar level in both strains PH-1::RP27-GFP and *dim2*::RP27-GFP, that of pRep(500)-GFP was increased greatly in strain *dim2*::pRep(500)-GFP versus PH-1::pRep(500)-GFP (Fig. 3B and C). This result was further confirmed by RT-qPCR analysis, which showed that corresponding GFP mRNA levels in *dim2*::pRep(500)-GFP were significantly increased compared to PH-1::pRep(500)-GFP (Fig. 3D). Next, we performed bisulfite sequencing to detect the methylation of the Rep promoter at high resolution. The methylation level of the Rep promoter was significantly lower in *dim2*::pRep(500)-GFP than in PH-1::pRep(500)-GFP (Fig. 3E and Supplementary Fig. S4A).

To investigate whether FgGMTV1 infection enhances the methylation level of the integrated pRep(500)-GFP, we introduced the infectious FgGMTV1 clone into PH-1::pRep(500)-GFP and *dim2*::pRep(500)-GFP strains through PEG-mediated protoplast transfection (Supplementary Fig. S5A). Southern hybridization analysis confirmed successful viral infection in all transfectants (Supplementary Fig. S5B). Comparative analysis revealed a significant reduction in green fluorescence intensity between FgGMTV1-infected strains and their virus-free counterparts (Supplementary Fig. S5A), a finding corroborated by RT-qPCR showing corresponding decreases in GFP mRNA levels following viral infection (Supplementary Fig. S5C). Next, we performed bisulfite sequencing to detect the methylation of the Rep promoter in viral and nuclear genomes. Compared to FgGMTV1-infected PH-1::pRep(500)-GFP, the Rep promoter in viral genome showed lower methylation in FgGMTV1-infected *dim2*::pRep(500)-GFP (Supplementary Fig. S4B and S5D). Then, the methylation level of the Rep promoter in nuclear genome was significantly higher in FgGMTV1-infected PH-1::pRep(500)-GFP than in virus-free PH-1::pRep(500)-GFP, while *dim2*::pRep(500)-GFP strains maintained consistent methylation levels regardless of infection status (Supplementary Fig. S4C and S5E). These results suggest that DIM2-mediated methylation suppresses the FgGMTV1 Rep promoter-dependent transcriptional activity.

### DCLs and AGOs are involved in the DNA methylation of FgGMTV1

**Figure 4.**
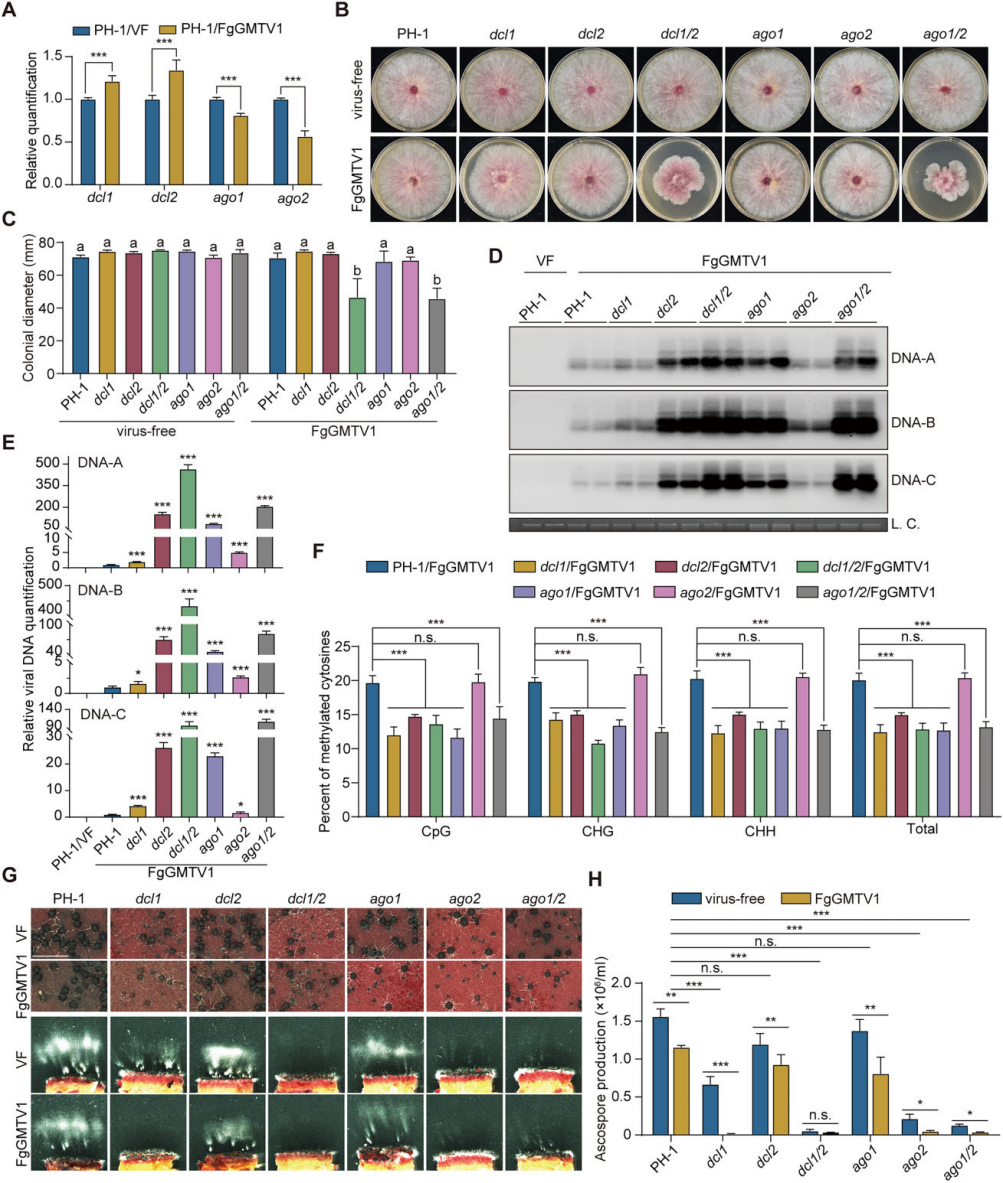
DCLs and AGOs are involved in FgGMTV1 DNA methylation. (**A**) Relative expression levels of *dcls* and *agos* in virus-free and FgGMTV1-infected strains assayed by qRT-PCR with RNA isolated from 4-day-old PDA cultures. (**B**) Colony morphologies of virus-free (top) and FgGMTV1-infected (bottom) mutant strains. (**C**) Colonial diameters of virus-free and FgGMTV1-infected mutant strains measured with 4-day-old PDA cultures. Mean and standard deviations were estimated with data from three independent replicates. Different letters indicate significant differences according to one-way ANOVA followed by Dunnett’s post-hoc test (*P* < 0.05). (**D**) Southern blots of genomic DNA isolated from 4-day-old PDA cultures in the dark were hybridized with probes A, B, and C that are specific for DNA-A, DNA-B, and DNA-C of FgGMTV1, respectively. LC, loading control to show equal amounts of genomic DNA in each lane. (**E**) The abundance of DNA-A, DNA-B, and DNA-C of FgGMTV1 in the same samples was assayed by qPCR. The *FgEF-1*α gene was used as the internal control. (**F**) Percentage of methylated cytosines in fragment MeA2 of FgGMTV1 in marked mutant strains determined by bisulfite sequencing. (**G**) Perithecium formation and ascospore discharge in mating cultures of the virus-free and FgGMTV1-infected marked mutant strains on carrot agar plates. Bar = 0.5 mm. (**H**) Number of the discharge ascospores produced by virus-free and FgGMTV1-infected marked mutant strains on carrot agar plates. For **A**, **E**, **F**, and **H**, values are represented as the mean ± SD (*n* = 3 independent experiments). Statistical significance was determined using one-way ANOVA followed by Dunnett’s post-hoc test. *, *P* < 0.05; **, *P* < 0.01; ***, *P* < 0.001; and n.s., not significant.

*F. graminearum* contains two Dicer (DCL1 and DCL2) and two Argonaute (AGO1 and AGO2) proteins [53] that are orthologous to the DCLs and AGOs important for methylation and defense against DNA viruses in plants [13]. To investigate whether DCLs and AGOs are involved in FgGMTV1 infection in *F. graminearum*, the expression of *dcl* and *ago* genes was detected in both the virus-free and FgGMTV1-infected wild-type strain PH-1 by RT-qPCR. In the FgGMTV1-infected PH-1 strain, the expression of *dcl1* and *dcl2* was notably elevated compared to the virus-free PH-1 strain. In contrast, the expression of *ago1* and *ago2* exhibited a significant decrease (Fig. 4A).

To determine whether DCLs and AGOs are involved in DNA methylation of FgGMTV1, FgGMTV1 was introduced by the infectious clone of FgGMTV1 via PEG-mediated protoplast transfection into *F. graminearum* single gene deletion mutants (*dcl1*, *dcl2*, *ago1*, and *ago2*) and double-knockout mutants (*dcl1/2* and *ago1/2*) (Fig. 4B and Supplementary Fig. S6A–C). Southern blotting and qPCR analysis showed that the accumulation of FgGMTV1 in all *dcls* and *agos* deletion mutants was markedly higher than that of the FgGMTV1-infected PH-1 (Fig. 4D and E). Meanwhile, the accumulation of FgGMTV1 in double knockout mutants was significantly greater than in single knockout mutants, indicating that while DCL1 and AGO2 play a primary role in the sex-specific RNAi pathway [41], both members of the DCL and AGO families exhibit functional redundancies in the anti-FgGMTV1 response. Notably, the abundance of FgGMTV1 DNA was significantly elevated in FgGMTV1-infected *dcl2* and *ago1* mutants compared to *dcl1* and *ago2* mutants, suggesting that DCL2 and AGO1 play a pivotal role in the anti-FgGMTV1 pathway. Bisulfite sequencing results demonstrated significantly lower methylation in fragment MeA2 in FgGMTV1-infected strains of *dcl1*, *dcl2*, *dcl1/2*, *ago1*, and *ago1/2*, compared to the PH-1/FgGMTV1 strain (Fig. 4F and Supplementary Fig. S6D). These findings suggest that DCL1, DCL2, and AGO1 are involved in FgGMTV1 genome methylation.

Then, we evaluated colony morphology, mycelial growth rate, the responses to stress, virulence, and sexual/asexual reproduction of virus-free and FgGMTV1-infected *dcls* and *agos* deletion mutants. Compared to FgGMTV1-infected PH-1, abnormal colony morphology and severe growth retardation were observed in FgGMTV1-infected *dcl1/2* and *ago1/2* mutants (Fig. 4B and C). In virus-free strains, the deletion mutants of *dcls* and *agos* exhibited no significant alterations in SDS conditions compared to PH-1. However, upon infection with FgGMTV1, most of these mutants, with the exception of *ago2*, displayed elevated sensitivity to SDS compared to PH-1 (Supplementary Fig. S7A and B). Both in virus-free and FgGMTV1-infected conditions, the deletion mutants of *dcls* and *agos* showed increased sensitivity to H_2_O_2_ compared to PH-1 (Supplementary Fig. S7A and B). Additionally, when infected with FgGMTV1, the *dcl1*, *dcl1/2*, *ago1*, and *ago1/2* mutants exhibited further increased sensitivity to H_2_O_2_ compared to their virus-free counterparts (Supplementary Fig. S7A and B). In the virulence assay, the virulence of the FgGMTV1-infected *dcl1/2*, *ago1*, and *ago1/2* strains was significantly reduced compared to the wild-type strain on flowering wheat heads (Supplementary Fig. S7C and D). The deletion mutants of *dcls* and *agos*, with or without FgGMTV1, exhibited normal conidiation (Supplementary Fig. S7E). For sexual reproduction, the *dcl1*, *dcl1/2*, *ago1*, *ago2*, and *ago1/2* mutants formed markedly smaller perithecia, along with a decrease in ascospore formation and discharge (Fig. 4G and H). Furthermore, FgGMTV1-infection had an obvious effect on decreasing ascospore production in all mutants (Fig. 4G and H). These findings revealed that DCL1, DCL2, and AGO1 play crucial roles in the anti-FgGMTV1 pathway mediated by DNA methylation.

### DCLs and AGOs are involved in the RNAi-directed DNA methylation pathway of FgGMTV1

**Figure 5.**
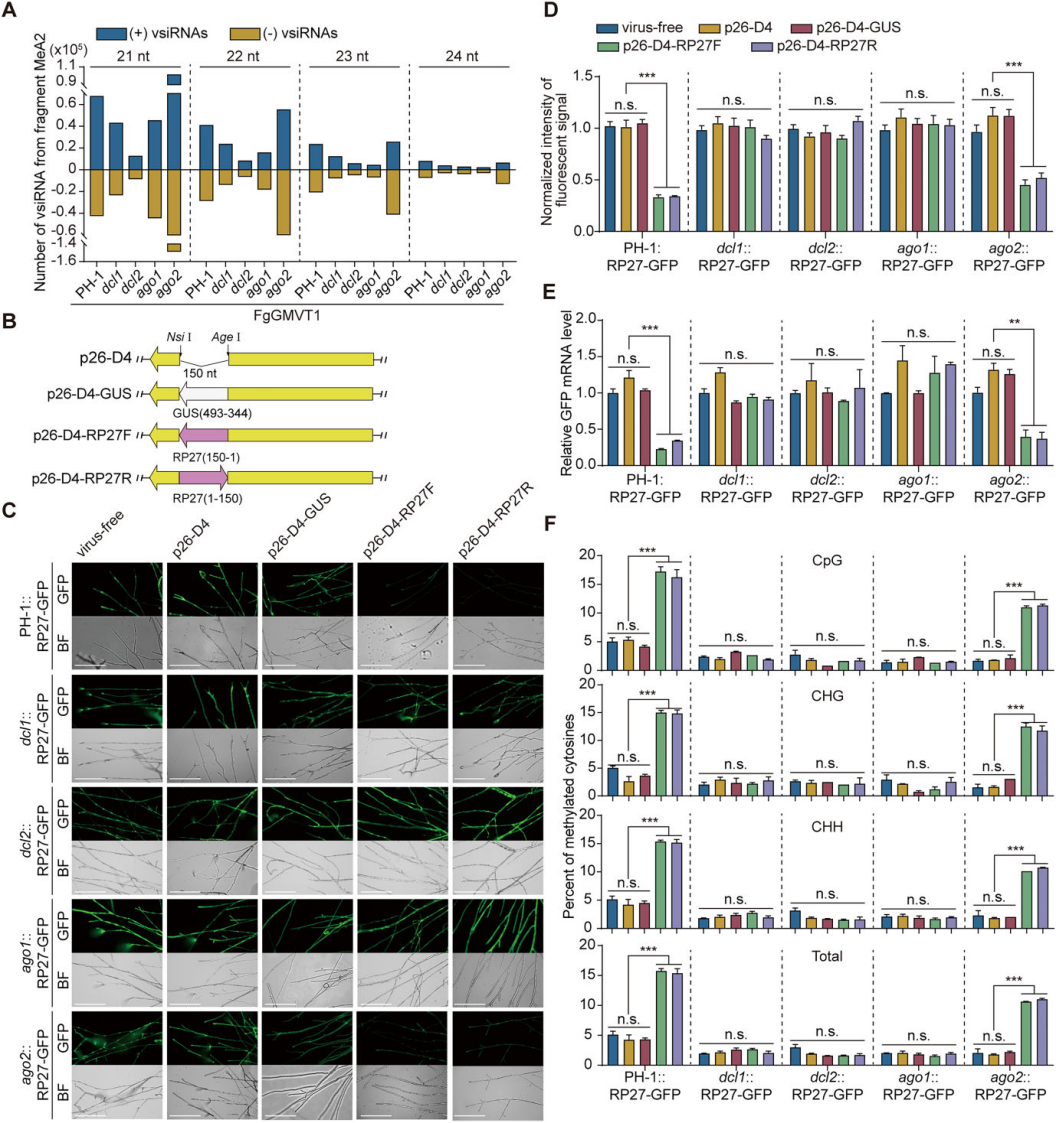
DCLs and AGOs are involved in the RNA-directed DNA methylation pathway of fungi. (**A**) Normalized read number of 21–24 nt vsiRNAs derived from fragment MeA2 in sRNA libraries of the FgGMTV1-infected mutant strains. “(−)” and “(+)” indicate the sRNAs derived from the complementary (negative) and positive viral genomic strands, respectively. (**B**) The p26 regions in the empty p26-D4 vector and p26-D4 with fragments of GUS and RP27 promoter sequences closed in its multiple cloning site (MCS). Yellow, white, and pink arrows indicate the orientations of p26, GUS, and RP27 promoter sequences, respectively. Numbers indicate the location of GUS and the RP27 promoter. (**C**) Representative images of marked strains that are virus free or infected with labeled viral constructs infected with no p26-D4-RP27F/R-infected mutant strains. Bar = 200 μm. (**D**) Normalized intensity of fluorescent signal in p26-D4-RP27F/R-infected mutant strains. The fluorescence intensity was quantified using the ImageJ software, with those in virus free strains set as 1.00. (**E**) Relative levels of GFP mRNA in p26-D4-RP27F/R-infected mutant strains. RT-qPCR was performed using the *FgEF-1α* transcript levels as the internal control. (**F**) Percentage of methylated cytosines in the RP27 promoter of p26-D4-RP27F/R-infected mutant strains determined via bisulfite sequencing. For **D**, **E**, and **F**, bars of the same color represent the same strain samples. values are represented as the mean ± SD (*n* = 3 independent experiments). Statistical significance was determined using one-way ANOVA followed by Dunnett’s post-hoc test. **, *P* < 0.01; ***, *P* < 0.001; and n.s., not significant.

In plants, DNA methylation is closely related to 21–24 nt sRNAs [54]. To investigate the relationship between FgGMTV1 DNA methylation and siRNAs produced by the RNAi machinery, we analyzed the association between the distribution of virus-derived siRNAs (vsiRNAs) and DNA methylation in the FgGMTV1 genome [39]. An enrichment hotspot of FgGMTV1-derived siRNAs was observed in the MeA2, MeB6, and MeC5 DNA methylation hotspot regions (Supplementary Fig. S8A–C). Total vsiRNAs were subjected to deep sequencing analysis in the FgGMTV1-infected PH-1, *dcl1*, *dcl2*, *ago1*, and *ago2* strains. Next, we analyzed the 21–24 nt vsiRNAs derived from the fragment MeA2 in all strains separately. The accumulation of 21–24 nt vsiRNAs derived from the fragment MeA2 in the FgGMTV1-infected *dcl1*, *dcl2*, and *ago1* mutants was significantly decreased respectively, compared to that in PH-1/FgGMTV1, but that in FgGMTV1-infected *ago2* was increased (Fig. 5A). Meanwhile, the 21–24 nt vsiRNAs derived from the fragment MeC5 showed a similar trend to that from the fragment MeA2 in the FgGMTV1-infected strains of *dcl1*, *dcl2*, and *ago1* (Supplementary Fig. S8D). This observation suggests that the low DNA methylation level of FgGMTV1-infected strains of *dcl1*, *dcl2*, and *ago1* in *F. graminearum* is correlated with a lower production of viral 21–24 nt siRNAs.

In plants, RdDM can be manipulated to target and silence some genes in a highly specific manner. For example, foreign genes expressed under the control of the cauliflower mosaic virus (CaMV) 35S promoter are silenced by inducing hypermethylation of the 35S promoter following infection by a virus carrying the same promoter sequence [55, 56]. To investigate whether there is a similar phenomenon in fungi, we used the FgGMTV1-based VIGS vector p26-D4 [35] to construct VIGS vectors of the RP27 promoter, p26-D4-RP27F and p26-D4-RP27R, which carried 150 bp sense and antisense sequences from the RP27 promoter (Fig. 5B). These vectors p26-D4 (empty VIGS vector), p26-D4-GUS (as a VIGS control), p26-D4-RP27F/R were transfected into the PH-1::RP27-GFP strain via PEG-mediated protoplast transfection (Supplementary Fig. S9A). Southern blotting analysis revealed that all the vectors were infective (Supplementary Fig. S9B). To verify the efficacy of gene silencing, we assessed the fluorescence intensity and transcriptional levels of GFP in p26-D4-RP27F/R infected PH-1::RP27-GFP strains. Compared with that in the virus-free, p26-D4, and p26-D4-GUS-infected PH-1::RP27-GFP strains, green fluorescence intensity was significantly reduced in the p26-D4-RP27F/R-infected PH-1::RP27-GFP strains (Fig. 5C and D). This result was further confirmed via RT-qPCR analysis, which showed that corresponding GFP mRNA levels were lower in the p26-D4-RP27F/R-infected PH-1::RP27-GFP strains than in the virus-free, p26-D4, and p26-D4-GUS-infected strains (Fig. 5E).

We investigated the methylation status of the RP27 promoter in p26-D4-RP27F/R-infected PH-1::RP27-GFP strains. Bisulfite sequencing analyzes revealed that the methylation level of the RP27 promoter was significantly higher in the p26-D4-RP27F/R-infected PH-1::RP27-GFP strains than in the virus-free, p26-D4, and p26-D4-GUS-infected strains (Fig. 5F and Supplementary Fig. S10). These findings revealed that small RNA from FgGMTV1 mediated DNA methylation suppresses integrated RP27 promoter-dependent transcriptional activity.

To test the role of DCLs and AGOs in the RNAi-directed DNA methylation pathway, we generated integrated RP27-GFP expressing strains of *dcl1*::RP27-GFP, *dcl2*::RP27-GFP, *ago1*::RP27-GFP, and *ago2*::RP27-GFP, and transfected p26-D4-RP27F/R into them (Supplementary Fig. S9A). Southern blot analysis indicated that all the vectors were infective (Supplementary Fig. S9B). The fluorescence intensity analysis demonstrated that no reduction of GFP fluorescence intensity was observed in the p26-D4-RP27F/R-infected *dcl1*::RP27-GFP, *dcl2*::RP27-GFP, and *ago1*::RP27-GFP strains, compared to the virus-free, p26-D4, and p26-D4-GUS-infected strains (Fig. 5C and D). The relative level of GFP mRNA in the p26-D4-RP27F/R-infected *dcl1*::RP27-GFP, *dcl2*::RP27-GFP, and *ago1*::RP27-GFP strains are similar for the virus-free, p26-D4, and p26-D4-GUS-infected strains (Fig. 5E). However, similar to the p26-D4-RP27F/R infected PH-1::RP27-GFP strains, the GFP fluorescence intensity and relative level of GFP mRNA in the p26-D4-RP27F/R-infected *ago2*::RP27-GFP strains were significantly reduced (Fig. 5C–E). The hypomethylation of the RP27 promoter in p26-D4-RP27F/R-infected *dcl1*::RP27-GFP, *dcl2*::RP27-GFP, and *ago1*::RP27-GFP strains were observed, and was similar to the virus-free, p26-D4, and p26-D4-GUS-infected strains (Fig. 5F and Supplementary Fig. S10). But the methylation level of the RP27 promoter was significantly higher in p26-D4-RP27F/R infected *ago2*::RP27-GFP strains than in virus-free, p26-D4 or p26-D4-GUS infected strains (Fig. 5F and Supplementary Fig. S10). Taken together, these results suggest that DCL1, DCL2, and AGO1 are involved in the RNAi-directed DNA methylation pathway in fungi.

## Discussion

### Evolutionary conservation of viral promoter methylation in eukaryotic antiviral defense

DNA methylation-mediated TGS is a well-characterized defense mechanism against geminiviruses in plants [13]. These viruses replicate through dsDNA intermediates that assemble into nucleosomes, rendering them ideal substrates for methylation [38]. For example, mutants of *Arabidopsis* with deficient DNA methylation (e.g. *cmt3*, *drm2*, and *ago4*) exhibit increased sensitivity to CaMV and bean common mosaic virus, accompanied by reduced viral genome methylation [13]. Our findings extend this paradigm to the fungal kingdom through the characterization of *F. graminearum*’s DIM2-dependent methylation system targeting mycovirus FgGMTV1 (Fig. 2). Bisulfite sequencing revealed hypermethylation hotspots within viral promoter regions—specifically fragment MeA2 (78–355 nt) in DNA-A (Rep promoter), MeC5 (748–1125 nt) in DNA-C (p26 promoter), and MeC1 (1170–113 nt) governing p9/p18 expression [37] (Fig. 1). These findings mirror observations in *Nicotiana benthamiana* infected with tomato yellow leaf curl China virus (TYLCCNV), where promoter-adjacent hypermethylation occurs [57], suggesting promoter-targeted DNA methylation represents a universal eukaryotic defense mechanism against DNA viruses.

Viral promoter methylation typically suppresses transcription by either blocking transcription factor binding or inducing chromatin modifications [58, 59]. TLCV has been demonstrated to induce TGS of a homologous integrated plant transgene, which is characterized by extensive hypermethylation- of the virus-derived sequences within the transgenic locus [17]. To dissect this mechanism, we engineered transgenic strains with GFP expression driven by the Rep promoter. *dim2* deletion reduced promoter methylation while increasing GFP expression (Fig. 3). Conversely, introducing p26-D4-RP27 triggered RP27 promoter hypermethylation and GFP silencing (Fig. 5). These results demonstrate that fungal viral infections generate sequence-specific methylation signals inducing TGS. Although DNA methylation is tightly linked to transcriptional repression, the underlying molecular machinery remains elusive in fungi. In plants, methyl-binding proteins (MBPs) recruit repressive complexes (e.g. HDACs, polycomb proteins) through distinct domains (MBD or Kaiso/BTB zinc fingers) [60, 61]. The absence of fungal MBP homologs suggests divergent silencing mechanisms, establishing our system as a unique platform to explore fungal-specific TGS pathways.

### RdDM as an ancient and conserved regulatory strategy across eukaryotes

While RNAi-mediated DNA methylation was first described in plants as early as 1994 [62, 63], no fungal counterparts had been reported until now. Our work provides the first evidence of RdDM in fungi, resolving longstanding questions about RNAi-DNA methylation crosstalk in this kingdom. Plant canonical RdDM involves Pol IV/RDR2-dependent dsRNA synthesis, DCL3 processing into 24-nt sRNAs, and AGO4/6/9-guided DRM2 recruitment [23]. While Pol IV and Pol V are plant-specific multi-subunit RNA polymerases, they both evolved from Pol II, which is shared by eukaryotes [64, 65]. Pol II is supposed to serve as an enzyme assisting in the targeting of RdDM or RNA-directed histone modifications in invertebrates and yeasts, which lack specialised RdDM polymerases [66]. Intriguingly, FgGMTV1-derived vsiRNAs (21–22 nt) correlated spatially with methylation hotspots, with minimal 24-nt species detected (Fig. 5 and S7). This contrasts with canonical plant RdDM but aligns with non-canonical RdDM-associated pathways [23]. These findings not only illuminate fundamental epigenetic mechanisms in fungi but also imply that RNAi-directed DNA methylation may represent an ancient and conserved regulatory strategy across eukaryotes.

The functional repertoire of fungal DNMTs and RNAi components varies considerably across species. The fungal kingdom exhibits remarkable diversity in DNMTs, with major genotypes including DNMT1 + DNMT5, DIM2 + DNMT5 + RID, and DNMT1 [12]. While RNAi pathways are essential for regulating vegetative growth and conidiation in *Cryptococcus neoformans, N. crassa*, *Mucor circinelloides*, and *Magnaporthe oryzae* [67–70], *F. graminearum* RNAi-deficient mutants (*dcl1/2* and *ago1/2*) exhibit no discernible defects in these developmental processes (Fig. 4). In addition, RNAi is essential for antiviral defense in *C. parasitica* [71] and *F. graminearum* (Fig. 4). Given the diversity of DCLs, AGOs, and DNMTs across fungi, further research is needed to elucidate the conservation and divergence of RNAi-mediated DNA methylation in other fungal species.

### Integrated antiviral strategies: balancing immediate and sustained responses

Our dissection of *F. graminearum*’s defense architecture reveals a sophisticated layering of antiviral mechanisms. DCL2 and AGO1 play dual roles in antiviral defense mechanisms, contributing not only to RNAi-mediated DNA methylation but also to PTGS in *F. graminearum* [72, 73]. Our study reveals that *dim2* deletion exerts a more pronounced negative effect on FgGMTV1 Rep promoter methylation compared to disruption of key RNAi components (Fig. 2F and 4F). Notably, while *dim2* knockout only moderately increases viral load (approximately 2-fold), RNAi mutations result in substantially greater viral accumulation (>10-fold), indicating that PTGS functions as the principal antiviral defense mechanism, with RdDM operating as a secondary regulatory strategy. This functional hierarchy resembles transitional models proposed in plants, where RNAi signals bridge acute PTGS responses to persistent TGS states [23].

Notably, residual DNA methylation persists in mutants lacking *dim2*, *dcls*, or *agos*, suggesting the existence of methylation pathways independent of both DIM2 and the RNAi machinery. This observation parallels findings in *N. crassa*, where DNA methylation is associated with RNAi-independent processes such as RID-mediated RIP and DIM5-dependent H3K9 methylation [6, 7]. These collective findings propose that *F. graminearum* likely harbors an alternative RNAi-independent DNA methylation pathway—a hypothesis demanding systematic investigation.

## Conclusion and perspectives

Beyond advancing fundamental mycovirology, our findings establish a pioneering framework for RNA-guided epigenetic therapeutics. While current RNA-based approaches predominantly focus on targeting RNA molecules through antisense oligonucleotides or RNAi triggers [74], we demonstrate a paradigm-shifting capability: sequence-specific sRNAs can direct locus-specific DNA methylation via RdDM mechanisms in fungi. This breakthrough provides proof-of-concept for engineering synthetic “epigenetic RNAs” capable of programming precise DNA methylation patterns, opening transformative avenues including the development of sequence-specific DNA methyltransferases guided by designer RNAs, the engineering of cross-kingdom epigenetic regulation systems, and the creation of RNA-based chromatin editors with broad agricultural and biomedical applications. Future investigations should prioritize resolving key mechanistic questions, including the functional role of RNA polymerase II in fungal RdDM compared to plant-specific Pol IV/V systems, and structural determinants governing DIM2 targeting by AGO1-vsiRNA complexes. Given this phylogenetic conservation of RdDM across plants and fungi, intriguing questions arise about its potential role in animals and humans. Addressing these knowledge gaps will be essential for translating this epigenetic programming platform into practical technologies.

In summary, by establishing *F. graminearum* as a model for fungal epigenetic virology, this work bridges critical gaps in understanding eukaryotic antiviral strategies. The discovery of RNAi-dependent DNA methylation challenges existing paradigms of fungal epigenetics while revealing deep evolutionary connections across eukaryotes. These insights advance our understanding of host-virus coevolution and catalyze innovations in RNA-based epigenetic engineering.

## Supplementary Material

gkaf478_Supplemental_File

## Data Availability

sRNA-seq files are accessible through the NCBI Sequence Read Archive (SRA) accession number PRJNA1212845 and as part of previously reported series PRJNA884307 [39].

## References

[1] Martienssen RA, Colot V DNA methylation and epigenetic inheritance in plants and filamentous fungi. Science. 2001; 293:1070–4. 10.1126/science.293.5532.1070. 11498574

[2] Zhang HM, Lang ZB, Zhu JK Dynamics and function of DNA methylation in plants. Nat Rev Mol Cell Biol. 2018; 19:489–506. 10.1038/s41580-018-0016-z. 29784956

[3] Schubeler D Function and information content of DNA methylation. Nature. 2015; 517:321–6. 10.1038/nature14192. 25592537

[4] Madhani HD Unbelievable but true: epigenetics and chromatin in fungi. Trends Genet. 2021; 37:12–20. 10.1016/j.tig.2020.09.016. 33092902 PMC8994648

[5] Miao VP, Freitag M, Selker EU Short TpA-rich segments of the ζ-η region induce DNA methylation in *Neurospora crassa*. J Mol Biol. 2000; 300:249–73. 10.1006/jmbi.2000.3864. 10873464

[6] Tamaru H, Selker EU A histone H3 methyltransferase controls DNA methylation in *Neurospora crassa*. Nature. 2001; 414:277–83. 10.1038/35104508. 11713521

[7] Freitag M, Williams RL, Kothe GO et al. A cytosine methyltransferase homologue is essential for repeat-induced point mutation in *Neurospora crassa*. Proc Natl Acad Sci USA. 2002; 99:8802–7. 10.1073/pnas.132212899. 12072568 PMC124379

[8] Kouzminova E, Selker EU *dim-2* encodes a DNA methyltransferase responsible for all known cytosine methylation in *Neurospora*. EMBO J. 2001; 20:4309–23. 10.1093/emboj/20.15.4309. 11483533 PMC149169

[9] Rossignol JL, Faugeron G MIP: an epigenetic gene silencing process in *Ascobolus immersus*. Curr Top Microbiol Immunol. 1995; 197:179–91. 7493492 10.1007/978-3-642-79145-1_12

[10] Malagnac F, Wendel B, Goyon C et al. A gene essential for *de novo* methylation and development in *Ascobolus* reveals a novel type of eukaryotic DNA methyltransferase structure. Cell. 1997; 91:281–90. 10.1016/S0092-8674(00)80410-9. 9346245

[11] Grognet P, Timpano H, Carlier F et al. A RID-like putative cytosine methyltransferase homologue controls sexual development in the fungus *Podospora anserina*. PLoS Genet. 2019; 15:e1008086 10.1371/journal.pgen.1008086. 31412020 PMC6709928

[12] Bewick AJ, Hofmeister BT, Powers RA et al. Diversity of cytosine methylation across the fungal tree of life. Nat Ecol Evol. 2019; 3:479–90. 10.1038/s41559-019-0810-9. 30778188 PMC6533610

[13] Raja P, Sanville BC, Buchmann RC et al. Viral genome methylation as an epigenetic defense against geminiviruses. J Virol. 2008; 82:8997–9007. 10.1128/JVI.00719-08. 18596098 PMC2546898

[14] Milavetz BI, Balakrishnan L Viral epigenetics. Methods Mol Biol. 2015; 1238:569–96. 10.1007/978-1-4939-1804-1_30. 25421681 PMC4478594

[15] Zarreen F, Chakraborty S Epigenetic regulation of geminivirus pathogenesis: a case of relentless recalibration of defence responses in plants. J Exp Bot. 2020; 71:6890–906. 10.1093/jxb/eraa406. 32869846

[16] Butterbach P, Verlaan MG, Dullemans A et al. Tomato yellow leaf curl virus resistance by *Ty-1* involves increased cytosine methylation of viral genomes and is compromised by cucumber mosaic virus infection. Proc Natl Acad Sci USA. 2014; 111:12942–7. 10.1073/pnas.1400894111. 25136118 PMC4156758

[17] Seemanpillai M, Dry I, Randles J et al. Transcriptional silencing of geminiviral promoter-driven transgenes following homologous virus infection. MPMI. 2003; 16:429–38. 10.1094/MPMI.2003.16.5.429. 12744514

[18] Hoelzer K, Shackelton LA, Parrish CR Presence and role of cytosine methylation in DNA viruses of animals. Nucleic Acids Res. 2008; 36:2825–37. 10.1093/nar/gkn121. 18367473 PMC2396429

[19] Ayllón MA, Vainio EJ Mycoviruses as a part of the global virome: diversity, evolutionary links and lifestyle. Adv Virus Res. 2023; 115:1–86. 10.1016/bs.aivir.2023.02.002. 37173063

[20] Martienssen R, Moazed D RNAi and heterochromatin assembly. Cold Spring Harb Perspect Biol. 2015; 7:a019323 10.1101/cshperspect.a019323. 26238358 PMC4526745

[21] Matzke MA, Mosher RA RNA-directed DNA methylation: an epigenetic pathway of increasing complexity. Nat Rev Genet. 2014; 15:394–408. 10.1038/nrg3683. 24805120

[22] Castel SE, Martienssen RA RNA interference in the nucleus: roles for small RNAs in transcription, epigenetics and beyond. Nat Rev Genet. 2013; 14:100–12. 10.1038/nrg3355. 23329111 PMC4205957

[23] Erdmann RM, Picard CL RNA-directed DNA methylation. PLoS Genet. 2020; 16:e1009034 10.1371/journal.pgen.1009034. 33031395 PMC7544125

[24] Siomi MC, Sato K, Pezic D et al. PIWI-interacting small RNAs: the vanguard of genome defence. Nat Rev Mol Cell Biol. 2011; 12:246–58. 10.1038/nrm3089. 21427766

[25] Ernst C, Odom DT, Kutter C The emergence of piRNAs against transposon invasion to preserve mammalian genome integrity. Nat Commun. 2017; 8:1411 10.1038/s41467-017-01049-7. 29127279 PMC5681665

[26] Watanabe T, Tomizawa S, Mitsuya K et al. Role for piRNAs and noncoding RNA in *de novo* DNA methylation of the imprinted mouse Rasgrf1 locus. Science. 2011; 332:848–52. 10.1126/science.1203919. 21566194 PMC3368507

[27] Volpe TA, Kidner C, Hall IM et al. Regulation of heterochromatic silencing and histone H3 lysine-9 methylation by RNAi. Science. 2002; 297:1833–7. 10.1126/science.1074973. 12193640

[28] Freitag M, Lee DW, Kothe GO et al. DNA methylation is independent of RNA interference in *Neurospora*. Science. 2004; 304:1939 10.1126/science.1099709. 15218142

[29] Groth M, Stroud H, Feng S et al. SNF2 chromatin remodeler-family proteins FRG1 and -2 are required for RNA-directed DNA methylation. Proc Natl Acad Sci USA. 2014; 111:17666–71. 10.1073/pnas.1420515111. 25425661 PMC4267348

[30] Catania S, Dumesic PA, Pimentel H et al. Evolutionary persistence of DNA methylation for millions of years after ancient loss of a *de novo* methyltransferase. Cell. 2020; 180:263–77. 10.1016/j.cell.2019.12.012. 31955845 PMC7197499

[31] Nai YS, Huang YC, Yen MR et al. Diversity of fungal DNA methyltransferases and their association with DNA methylation patterns. Front Microbiol. 2021; 11:616922 10.3389/fmicb.2020.616922. 33552027 PMC7862722

[32] Huang RR, Ding QQ, Xiang YN et al. Comparative analysis of DNA methyltransferase gene family in fungi: a focus on *basidiomycota*. Front Plant Sci. 2016; 7:1556 10.3389/fpls.2016.01556. 27818666 PMC5073141

[33] Zhang Z, Wen J, Li J et al. The evolution of genomic and epigenomic features in two *Pleurotus* fungi. Sci Rep. 2018; 8:8313 10.1038/s41598-018-26619-7. 29844491 PMC5974365

[34] Dean R, Van Kan JA, Pretorius ZA et al. The top 10 fungal pathogens in molecular plant pathology. Mol Plant Pathol. 2012; 13:414–30. 10.1111/j.1364-3703.2011.00783.x. 22471698 PMC6638784

[35] Chen Y, Kistler HC, Ma ZH *Fusarium graminearum* trichothecene mycotoxins: biosynthesis, regulation, and management. Annu Rev Phytopathol. 2019; 57:15–39. 10.1146/annurev-phyto-082718-100318. 30893009

[36] Li P, Wang S, Zhang L et al. A tripartite ssDNA mycovirus from a plant pathogenic fungus is infectious as cloned DNA and purified virions. Sci Adv. 2020; 6:eaay9634 10.1126/sciadv.aay9634. 32284975 PMC7138691

[37] Zhang L, Li P, Wang Y et al. p18 encoded by FgGMTV1 is responsible for asymptomatic infection in *Fusarium graminearum*. mBio. 2024; 25:e0306624. 10.1128/mbio.03066-24 PMC11708013 39584833

[38] Hanley-Bowdoin L, Settlage S, Orozco BM et al. Geminiviruses: models for plant DNA replication, transcription, and cell cycle regulation. Crit Rev Plant Sci. 1999; 18:71–106. 10.1080/07352689991309162. 10821479

[39] Wang S, Ruan S, Zhang M et al. Interference of small RNAs in *Fusarium graminearum* through FgGMTV1 infection. J Fungi. 2022; 8:1237 10.3390/jof8121237. PMC9781238 36547570

[40] Zhang L, Wang S, Ruan S et al. A mycovirus VIGS vector confers hypovirulence to a plant pathogenic fungus to control wheat FHB. Adv Sci. 2023; 10:e2302606 10.1002/advs.202302606. PMC10582431 37587761

[41] Son H, Park AR, Lim JY et al. Genome-wide exonic small interference RNA-mediated gene silencing regulates sexual reproduction in the homothallic fungus *Fusarium graminearum*. PLoS Genet. 2017; 13:e1006595 10.1371/journal.pgen.1006595. 28146558 PMC5310905

[42] Cuomo CA, Güldener U, Xu JR et al. The *Fusarium graminearum* genome reveals a link between localized polymorphism and pathogen specialization. Science. 2007; 317:1400–2. 10.1126/science.1143708. 17823352

[43] Sun M, Bian Z, Luan Q et al. Stage-specific regulation of purine metabolism during infectious growth and sexual reproduction in *Fusarium graminearum*. New Phytol. 2021; 230:757–73. 10.1111/nph.17170. 33411336

[44] Cao S, He Y, Hao C et al. RNA editing of the *AMD1* gene is important for ascus maturation and ascospore discharge in *Fusarium graminearum*. Sci Rep. 2017; 7:4617 10.1038/s41598-017-04960-7. 28676631 PMC5496914

[45] Yu JH, Hamari Z, Han KH et al. Double-joint PCR: a PCR-based molecular tool for gene manipulations in filamentous fungi. Fung Genet Biol. 2004; 41:973–81. 10.1016/j.fgb.2004.08.001. 15465386

[46] Zeng W, Wang J, Wang Y et al. Dicer-like proteins regulate sexual development via the biogenesis of perithecium-specific microRNAs in a plant pathogenic fungus *Fusarium graminearum*. Front Microbiol. 2018; 9:818 10.3389/fmicb.2018.00818. 29755439 PMC5932338

[47] Schneider CA, Rasband WS, Eliceiri KW NIH image to imageJ: 25 years of image analysis. Nat Methods. 2012; 9:671–5. 10.1038/nmeth.2089. 22930834 PMC5554542

[48] Hetzl J, Foerster AM, Raidl G et al. CyMATE: a new tool for methylation analysis of plant genomic DNA after bisulphite sequencing. Plant J. 2007; 51:526–36. 10.1111/j.1365-313X.2007.03152.x. 17559516

[49] Bonner C, Sproule A, Rowland O et al. DNA methylation is responsive to the environment and regulates the expression of biosynthetic gene clusters, metabolite production, and virulence in *Fusarium graminearum*. Front Fungal Biol. 2021; 1:614633 10.3389/ffunb.2020.614633. 37743878 PMC10512235

[50] Wang CF, Xu JR, Liu HQ A-to-I RNA editing independent of ADARs in filamentous fungi. RNA Biol. 2016; 13:940–5. 10.1080/15476286.2016.1215796. 27533598 PMC5056780

[51] Qi Z, Lu P, Long X et al. Adaptive advantages of restorative RNA editing in fungi for resolving survival-reproduction trade-offs. Sci Adv. 2024; 10:eadk6130 10.1126/sciadv.adk6130. 38181075 PMC10776026

[52] Ko YH, So KK, Chun J et al. Distinct roles of two DNA methyltransferases from *cryphonectria parasitica* in fungal virulence, responses to hypovirus infection, and viral clearance. mBio. 2021; 12:e02890-20 10.1128/mBio.02890-20. PMC8545091 33563819

[53] Chen Y, Gao Q, Huang M et al. Characterization of RNA silencing components in the plant pathogenic fungus *Fusarium graminearum*. Sci Rep. 2015; 5:12500 10.1038/srep12500. 26212591 PMC4515635

[54] Martins LM, Law JA Moving targets: mechanisms regulating siRNA production and DNA methylation during plant development. Curr Opin Plant Biol. 2023; 75:102435 10.1016/j.pbi.2023.102435. 37598540 PMC10581331

[55] Ruiz MT, Voinnet O, Baulcombe DC Initiation and maintenance of virus-induced gene silencing. Plant Cell. 1998; 10:937–46. 10.1105/tpc.10.6.937. 9634582 PMC144041

[56] Jones L, Hamilton AJ, Voinnet O et al. RNA-DNA interactions and DNA methylation in post-transcriptional gene silencing. Plant Cell. 1999; 11:2291–301. 10590159 10.1105/tpc.11.12.2291 PMC144133

[57] Yang X, Xie Y, Raja P et al. Suppression of methylation-mediated transcriptional gene silencing by bC1-SAHH protein interaction during geminivirus-betasatellite infection. PLoS Pathog. 2011; 7:e1002329 10.1371/journal.ppat.1002329. 22028660 PMC3197609

[58] Domcke S, Bardet AF, Adrian Ginno P et al. Competition between DNA methylation and transcription factors determines binding of NRF1. Nature. 2015; 528:575–9. 10.1038/nature16462. 26675734

[59] Zhu H, Wang G, Qian J Transcription factors as readers and effectors of DNA methylation. Nat Rev Genet. 2016; 17:551–65. 10.1038/nrg.2016.83. 27479905 PMC5559737

[60] Grimanelli D, Ingouff M DNA methylation readers in plants. J Mol Biol. 2020; 432:1706–17. 10.1016/j.jmb.2019.12.043. 31931004

[61] Ichino L, Boone BA, Strauskulage L et al. MBD5 and MBD6 couple DNA methylation to gene silencing through the J-domain protein SILENZIO. Science. 2021; 372:eabg6130. 10.1126/science.abg6130 PMC8639832 34083448

[62] Wassenegger M, Heimes S, Riedel L et al. RNA-directed *de novo* methylation of genomic sequences in plants. Cell. 1994; 76:567–76. 10.1016/0092-8674(94)90119-8. 8313476

[63] Nicolás FE, Torres-Martínez S, Ruiz-Vázquez RM Loss and retention of RNA interference in fungi and parasites. PLoS Pathog. 2013; 9:e1003089 10.1371/journal.ppat.1003089. 23358725 PMC3554617

[64] Haag JR, Pikaard CS Multisubunit RNA polymerases IV and V: purveyors of non-coding RNA for plant gene silencing. Nat Rev Mol Cell Biol. 2011; 12:483–92. 10.1038/nrm3152. 21779025

[65] Gallego-Bartolomé J, Liu W, Kuo PH et al. Co-targeting RNA polymerases IV and V promotes efficient *de novo* DNA methylation in *Arabidopsis*. Cell. 2019; 176:1068–82. 10.1016/j.cell.2019.01.029. 30739798 PMC6386582

[66] Johnson LM, Du J, Hale CJ et al. SRA- and SET-domain-containing proteins link RNA polymerase V occupancy to DNA methylation. Nature. 2014; 507:124–8. 10.1038/nature12931. 24463519 PMC3963826

[67] Janbon G, Maeng S, Yang DH et al. Characterizing the role of RNA silencing components in *Cryptococcus neoformans*. Fung Genet Biol. 2010; 47:1070–80. 10.1016/j.fgb.2010.10.005. PMC3021383 21067947

[68] Nolan T, Braccini L, Azzalin G et al. The post-transcriptional gene silencing machinery functions independently of DNA methylation to repress a LINE1-like retrotransposon in *Neurospora crassa*. Nucleic Acids Res. 2005; 33:1564–73. 10.1093/nar/gki300. 15767281 PMC1065258

[69] Nicolas FE, Moxon S, de Haro JP et al. Endogenous short RNAs generated by Dicer 2 and RNA-dependent RNA polymerase 1 regulate mRNAs in the basal fungus *Mucor circinelloides*. Nucleic Acids Res. 2010; 38:5535–41. 10.1093/nar/gkq301. 20427422 PMC2938224

[70] Nunes CC, Gowda M, Sailsbery J et al. Diverse and tissue-enriched small RNAs in the plant pathogenic fungus, *Magnaporthe oryzae*. BMC Genomics. 2011; 12:288 10.1186/1471-2164-12-288. 21635781 PMC3132168

[71] Segers GC, Zhang X, Deng F et al. Evidence that RNA silencing functions as an antiviral defense mechanism in fungi. Proc Natl Acad Sci USA. 2007; 104:12902–6. 10.1073/pnas.0702500104. 17646660 PMC1937564

[72] Yu J, Lee KM, Cho WK et al. Differential contribution of RNA interference components in response to distinct *Fusarium graminearum* virus infections. J Virol. 2018; 92:e01756–17. 10.1128/JVI.01756-17. 29437977 PMC5899199

[73] Yu J, Park JY, Heo JI et al. The ORF2 protein of fusarium graminearum virus 1 suppresses the transcription of *FgDICER2* and *FgAGO1* to limit host antiviral defences. Mol Plant Pathol. 2020; 21:230–43. 10.1111/mpp.12895. 31815356 PMC6988435

[74] Yu AM, Choi YH, Tu MJ RNA drugs and RNA targets for small molecules: principles, progress, and challenges. Pharmacol Rev. 2020; 72:862–98. 10.1124/pr.120.019554. 32929000 PMC7495341

