# Supplementary material for: Methylation of mycovirus DNA is mediated by the RNAi machinery in vegetative hyphae of Fusarium graminearum
Source: Nucleic Acids Res. 2025 Jun 16;53(11):gkaf478. doi: 10.1093/nar/gkaf478 (PMC12167651; doi:10.1093/nar/gkaf478)
Supplement: gkaf478_Supplemental_File [file gkaf478_supplemental_file.pdf]

Supporting Information for

Methylation of mycovirus DNA is mediated by the RNAi machinery in vegetative hyphae of *Fusarium graminearum*

Authors:
Yanfei Wang[1], Wei Chen[2], Lihang Zhang[1], Shuangchao Wang[1], Jin-Rong Xu[3], Lihua Guo[1]*

Affiliations:
[1]State Key Laboratory for Biology of Plant Diseases and Insect Pests, Institute of Plant Protection, Chinese Academy of Agricultural Sciences, Beijing, China.
[2]School of Life Science, Shanxi Normal University, Taiyuan, China.
[3]Department of Botany and Plant Pathology, Purdue University, West Lafayette, Indiana, USA.

* Correspondence: Lihua Guo, Institute of Plant Protection, Chinese Academy of Agricultural Sciences, Beijing, China, email: guolihua@caas.cn

Supplementary Figures

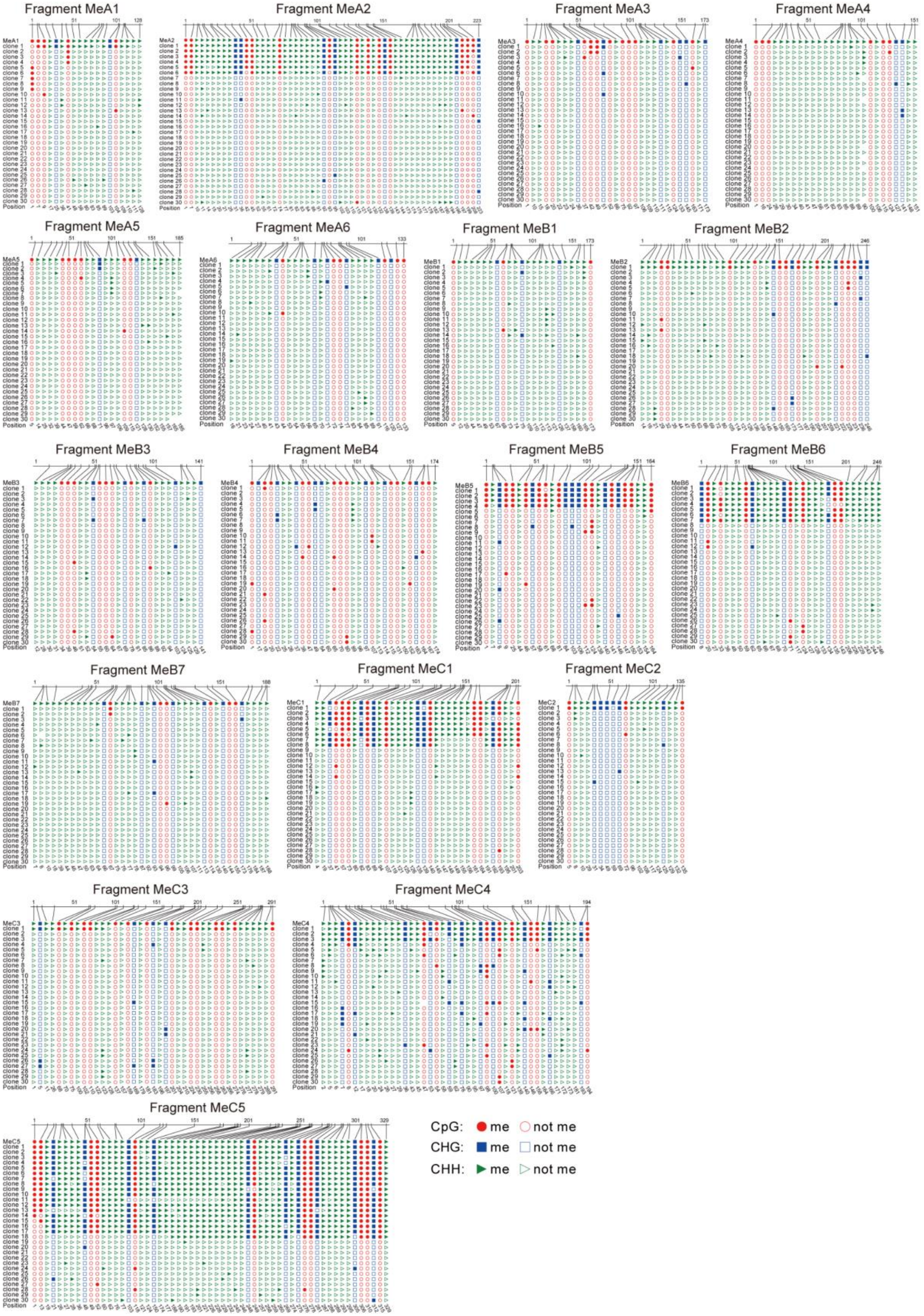

Figure S1 Bisulfite sequencing analysis of methylated cytosine at each fragment of FgGMTV1. The *F. graminearum* strain infected by FgGMTV1 was cultured on the PDA medium for 4 days at 25 °C. DNA was extracted, and the circular viral genome was divided into 5–7 fragments and amplified via PCR after bisulfite treatment. Thirty clones from each fragment were sequenced. Bisulfite sequencing data of individual clones were submitted to the CyMATE program (http://www.cymate.org) for the analysis of methylated cytosines. Circles indicate the cytosine residues and are color coded according to the sequence context (red for CG, blue for CHG, and green for CHH). Solid circles indicate the methylated cytosines. Each line indicates the sequence of an individual clone.

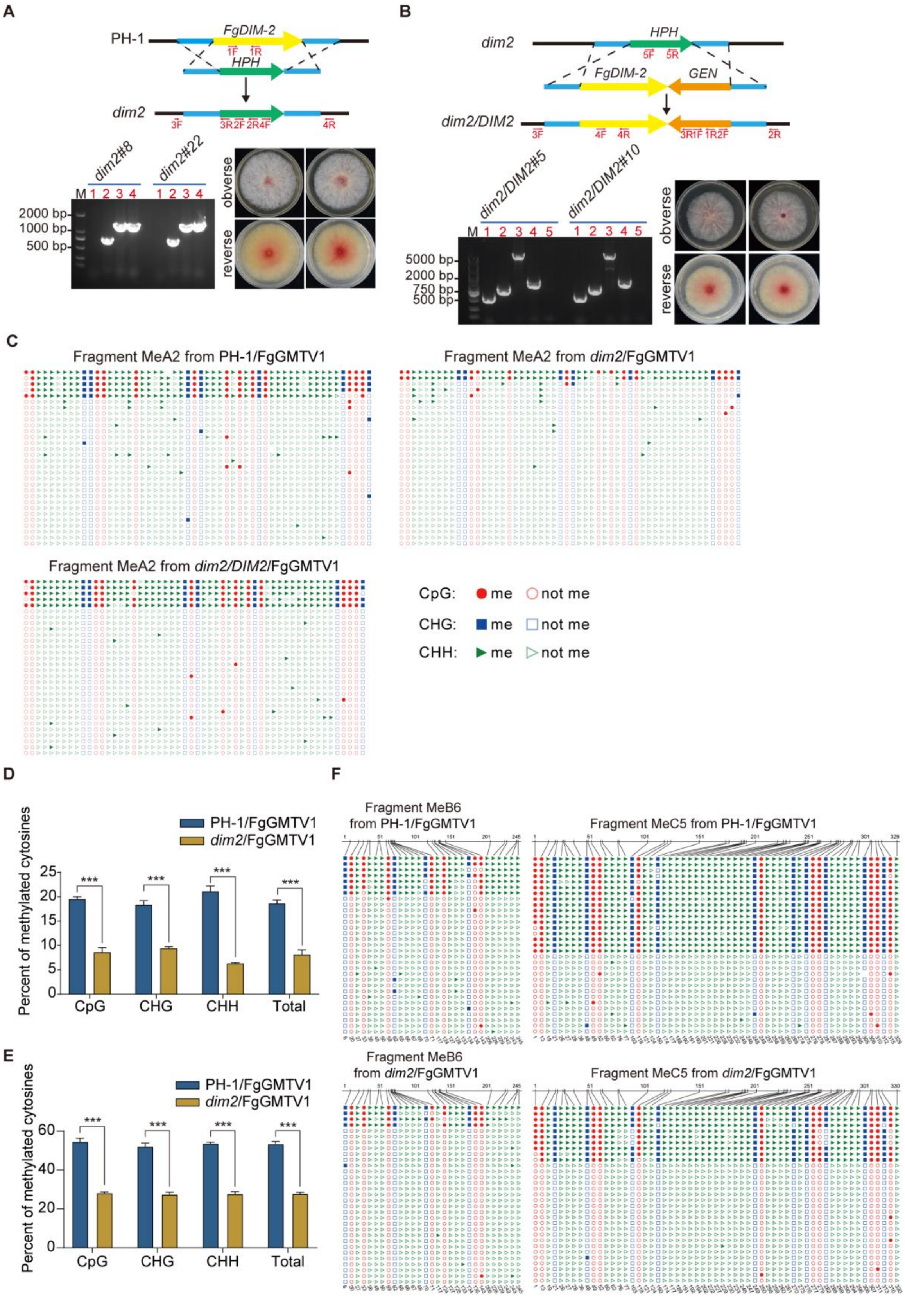
A
PH-1
FgDIM-2
1F
1R
HPH
dim2
3F
3R
2F
2R
4F
4R
dim2#8
dim2#22
M
1 2 3 4
1 2 3 4
2000 bp
1000 bp
500 bp
obverse
reverse
B
dim2
HPH
5F
5R
FgDIM-2
GEN
dim2/DIM2
3F
4F
4R
3R
1F
1R
2F
2R
dim2/DIM2#5
dim2/DIM2#10
M
1 2 3 4 5
1 2 3 4 5
5000 bp
2000 bp
750 bp
500 bp
obverse
reverse
C
Fragment MeA2 from PH-1/FgGMTV1
Fragment MeA2 from dim2/FgGMTV1
Fragment MeA2 from dim2/DIM2/FgGMTV1
CpG: me not me
CHG: me not me
CHH: me not me
D
PH-1/FgGMTV1
dim2/FgGMTV1
Percent of methylated cytosines
25
20
15
10
5
0
***
CpG
CHG
CHH
Total
E
PH-1/FgGMTV1
dim2/FgGMTV1
Percent of methylated cytosines
60
40
20
0
***
CpG
CHG
CHH
Total
F
Fragment MeB6 from PH-1/FgGMTV1
Fragment MeC5 from PH-1/FgGMTV1
Fragment MeB6 from dim2/FgGMTV1
Fragment MeC5 from dim2/FgGMTV1
1
51
101
151
201
245
251
301
329
330

Figure S2 Construction of the DNMT gene *dim2* deletion and complemented mutants and DNA methylation analysis in the fragments MeA2, MeB6, and MeC5 of FgGMTV1 in FgGMTV1-infected *dim2* deletion mutant. (**A**) The Construction strategy, PCR identification, and colony morphology of the *dim2* gene deletion mutant. Yellow arrow indicates the *dim2* gene sequence, green arrow indicates the *HPH* cassette, blue boxes indicate the upstream and downstream sequences of the *dim2* gene, and the red arrows indicate primer binding sites (see Table S1 for primer sequences). Confirmation of *dim2* gene deletion mutants by PCR. The mutants were identified using four pairs of primers (Table S1) which amplified the replaced gene (lane 1), the *HPH* gene (lane 2), and the upstream fragment (lane 3) and downstream fragment (lane 4) of the replaced gene. (**B**) The Construction strategy, PCR identification, and colony morphology of the *dim2* gene complemented strain. Orange arrow indicates the neomycin (NEO) resistance cassette. The strains were identified using five pairs of primers (Table S1) which amplified the *GEN* gene (lane 1), the upstream fragment (lane 3) and downstream fragment (lane 2) of the complemented gene, the complemented gene (lane 4), and the *HPH* gene (lane 5). (**C**) Cytosine methylation profiles of MeA2. Circles indicate the cytosine residues and are color coded according to the sequence context (red for CG, blue for CHG, and green for CHH). Solid circles indicate the methylated cytosines. Each line indicates the sequence of an individual clone. (**D-E**) Percentage of methylated cytosines in the fragments MeB6 (**D**) and MeC5 (**E**) of FgGMTV1 in the FgGMTV1-infected *dim2* deletion mutant after 4 days of culture on the PDA medium in the dark determined via bisulfite sequencing. Values are represented as the mean ± SD (n = 3 independent experiments). Statistical significance was determined using one-way ANOVA followed by Dunnett's post-hoc test. ***$p < 0.001$. (**F**) Cytosine methylation profiles of MeB6 and MeC5. Circles indicate the cytosine residues and are color coded according to the sequence context (red for CG, blue for CHG, and green for CHH). Solid circles indicate the methylated cytosines. Each line indicates the sequence of an individual clone.

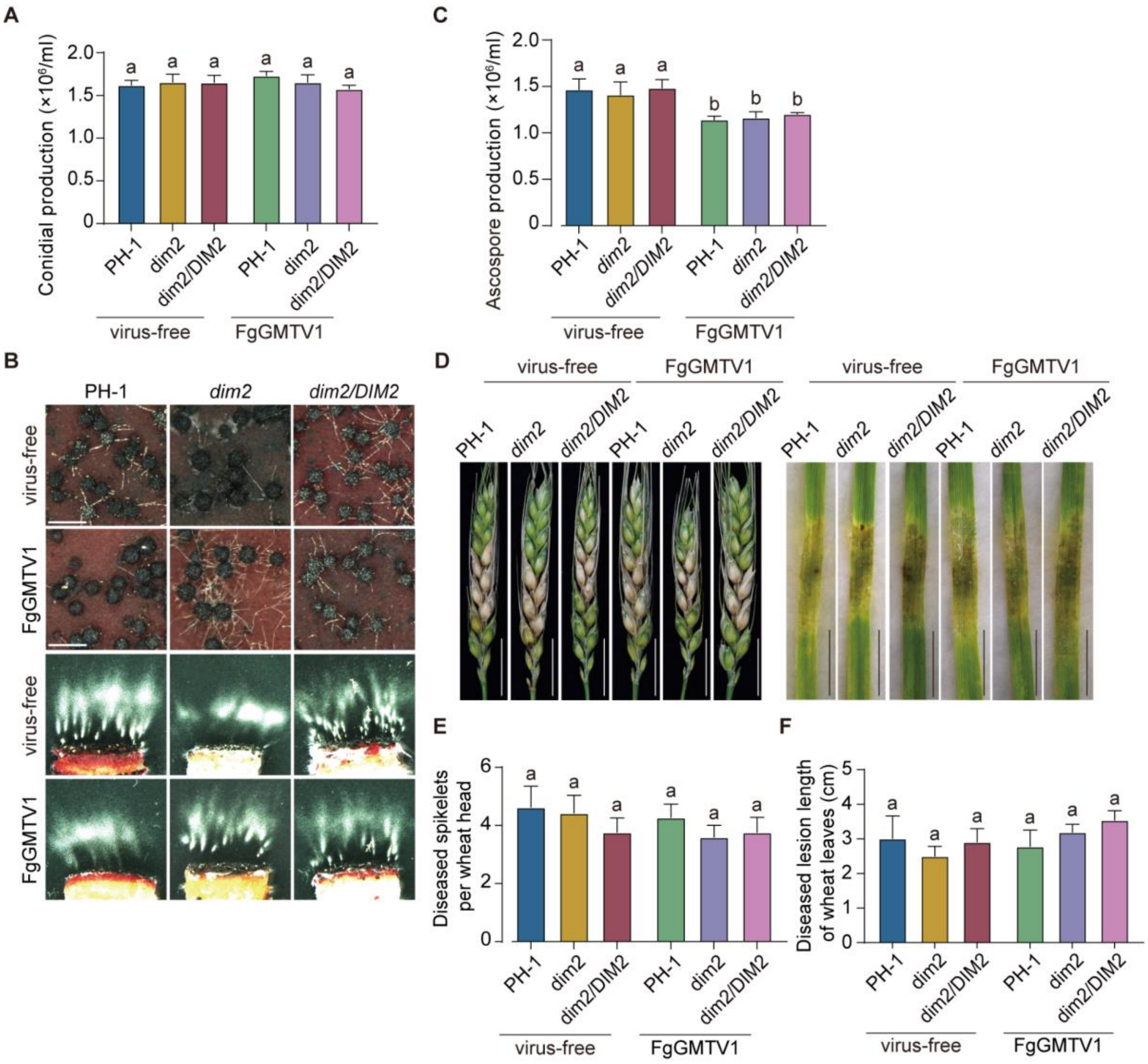

Figure S3 Characterization of virus-free and FgGMTV1-infected *dim2* deletion mutant. (**A**) Conidiation in 5-day-old CMC cultures of the virus-free and FgGMTV1-infected *dim2* deletion mutants. (**B**) perithecium formation and ascospore discharge in mating cultures of the virus-free and FgGMTV1-infected *dim2* deletion mutants on carrot agar plates. Bar = 0.5 mm. (**C**) Number of the discharge ascospores produced by virus-free and FgGMTV1-infected marked mutant strains on carrot agar plates. (**D**) Virulence of virus-free and FgGMTV1-infected *dim2* deletion mutant was evaluated on wheat heads and seedling leaves. Wheat heads inoculated with the indicated strains were examined for head blight symptoms at 14 days postinoculation (dpi). Wheat seedling leaves were inoculated with mycelial plugs and cultured at 28 °C for 7 dpi. Bar = 2 cm. (**E**) Number of diseased spikelets per invaded wheat head was counted at 14 dpi (n = 15). (**F**) The diseased lesion length of wheat leaves was analyzed at 7 dpi. For **A**, **C**, **E**, and **F**, values are represented as the mean ± SD (n = 3 independent experiments). Different letters indicate the statistically significant differences according to one-way ANOVA followed by Dunnett’s post-hoc test ($p < 0.05$).

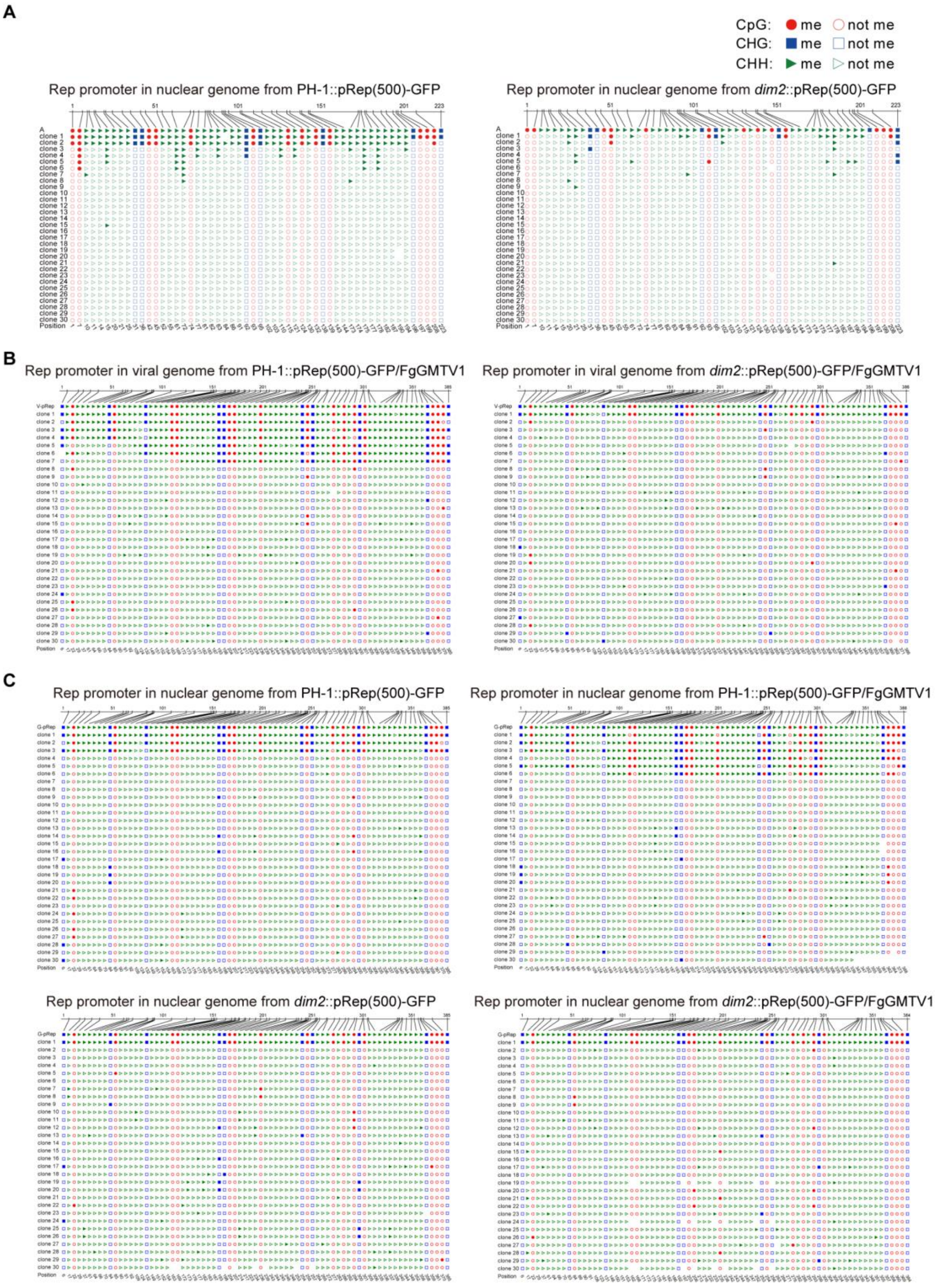

Figure S4 Cytosine methylation profiles of the Rep promoter in the viral and nuclear genomes in transgenic strains harboring pRep(500)-GFP cassettes. (**A**) Cytosine methylation profiles of the Rep promoter in the nuclear genome in the PH-1::pRep(500)-

GFP and *dim2*::pRep(500)-GFP strains. (**B**) Cytosine methylation profiles of the Rep promoter in the viral genome in the FgGMTV1-infected PH-1::pRep(500)-GFP and *dim2*::pRep(500)-GFP strains. (**C**) Cytosine methylation profiles of the Rep promoter in the nuclear genome in the virus-free and FgGMTV1-infected PH-1::pRep(500)-GFP and *dim2*::pRep(500)-GFP strains. All marked strains were cultured on the PDA medium for 4 days at 25 °C. The bisulfite sequencing data of individual clones were submitted to the CyMATE program (http://www.cymate.org) for analysis of the methylated cytosines. Circles indicate the cytosine residues and are color coded according to the sequence context (red for CG, blue for CHG, and green for CHH). Solid circles indicate the methylated cytosines. Each line indicates the sequence of an individual clone.

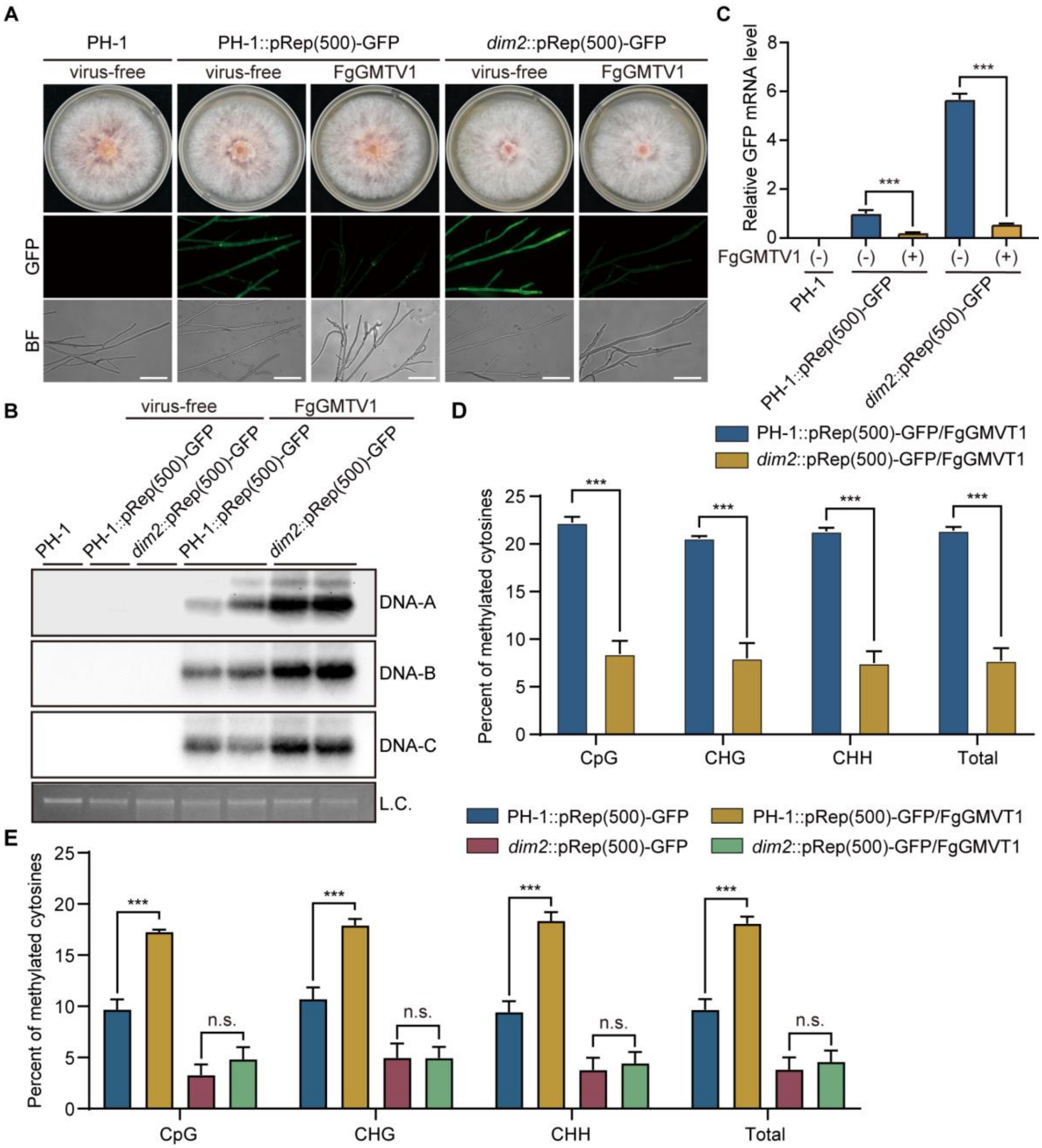

Figure S5 FgGMTV1 infection enhance methylation on the Rep promoter in the nuclear genomes. (**A**) Colony morphology and green fluorescence intensity of mycelia in the virus-free and FgGMTV1-infected PH-1 and *dim2* strains transformed with pRep(500)-GFP. All strains were cultured in the PDA medium for mycelia. Bar = 200 μm. (**B**) Southern blots of genomic DNA isolated from 4-day-old PDA cultures in the dark were hybridized with probes A, B, and C that are specific for DNA-A, DNA-B, and DNA-C of FgGMTV1, respectively. LC, loading control to show equal amounts of genomic DNA in each lane. (**C**) Normalized intensity of fluorescent signal in the virus-free and FgGMTV1-infected PH-1 and *dim2* strains transformed with pRep(500)-GFP. The fluorescence intensity was quantified using the ImageJ software, with those in the virus-free PH-1::pRep(500)-GFP strains set as 1.00. (**D**) Percentage of methylated cytosines in the Rep promoter of FgGMTV1 in FgGMTV1-infected PH-1::pRep(500)-GFP and

dim2::pRep(500)-GFP strains after 4 days of culture on the PDA medium in the dark determined by bisulfite sequencing. (**E**) Percentage of methylated cytosines in the Rep promoter in the nuclear genomes in the virus-free and FgGMTV1-infected PH-1::pRep(500)-GFP and *dim2*::pRep(500)-GFP strains after 4 days of culture on the PDA medium in the dark determined by bisulfite sequencing. For **C**, **D**, and **E**, values are represented as the mean ± SD (n = 3 independent experiments). Statistical significance was determined using one-way ANOVA followed by Dunnett's post-hoc test. ***$p < 0.001$ and n.s., not significant.

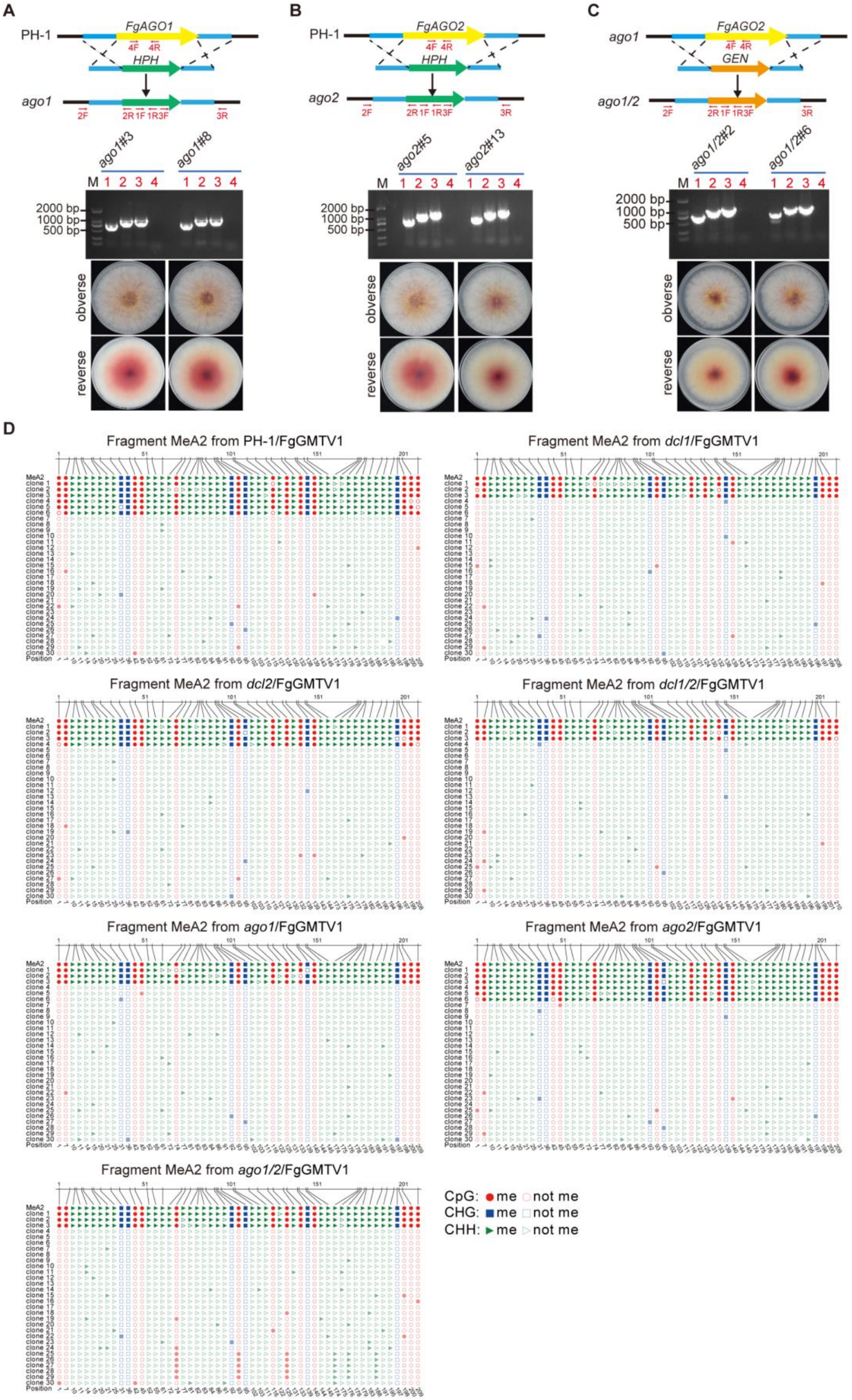
A
PH-1
FgAGO1
4F 4R
HPH
ago1
2F 2R 1F 1R 3F 3R
ago1#3
ago1#8
M 1 2 3 4 1 2 3 4
2000 bp
1000 bp
500 bp
obverse
reverse
B
PH-1
FgAGO2
HPH
ago2
ago2#5
ago2#13
C
ago1
FgAGO2
GEN
ago1/2
ago1/2#2
ago1/2#6
D
Fragment MeA2 from PH-1/FgGMTV1
Fragment MeA2 from dcl1/FgGMTV1
Fragment MeA2 from dcl2/FgGMTV1
Fragment MeA2 from dcl1/2/FgGMTV1
Fragment MeA2 from ago1/FgGMTV1
Fragment MeA2 from ago2/FgGMTV1
Fragment MeA2 from ago1/2/FgGMTV1
CpG: me not me
CHG: me not me
CHH: me not me

Figure S6 Construction of *ago1* and *ago2* single gene deletion and double-knockout mutants and bisulfite sequencing analysis of methylated cytosine at the fragment MeA2 in FgGMTV1-infected mutant strains. (**A**) The Construction strategy, PCR identification, and colony morphology of the *ago1* gene deletion mutant. (**B**) The Construction strategy, PCR identification, and colony morphology of *ago2* gene deletion mutant. (**C**) The Construction strategy, PCR identification, and colony morphology of *ago1* and *ago2* double-knockout mutant. Yellow arrow indicates the target gene, green arrow indicates the hygromycin resistance cassette (*HPH*), orange arrow indicates the geneticin resistance cassette (*GEN*), the blue boxes indicate the upstream and downstream sequences of the target gene, and the red arrows indicate primer binding sites (see Table S1 for primer sequences). Confirmation of gene deletion mutants via PCR. The mutants were identified using four pairs of primers (Table S1) which amplified the *HPH* gene (lane 1), the upstream fragment (lane 2) and downstream fragment (lane 3) of the replaced gene, and the replaced gene (lane 4). (**D**) Cytosine methylation profiles of MeA2 in FgGMTV1-infected mutants on PDA after incubation for 4 days. The bisulfite sequencing data of individual clones were submitted to the CyMATE program (http://www.cymate.org) for analysis of the methylated cytosines. Circles indicate the cytosine residues and are color coded according to the sequence context (red for CG, blue for CHG, and green for CHH). Solid circles indicate the methylated cytosines. Each line indicates the sequence of an individual clone.

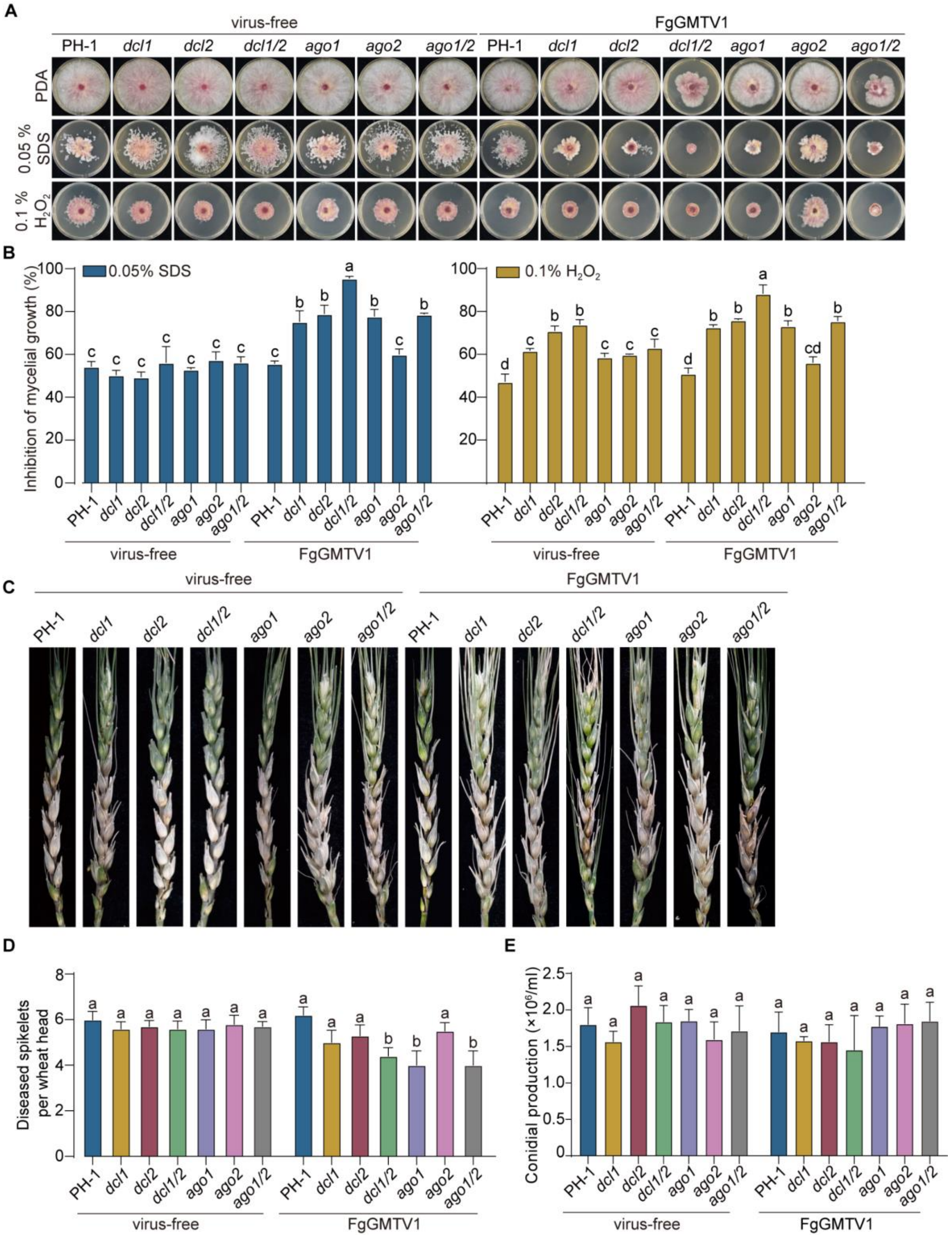

Figure S7 Characterization of virus-free and FgGMTV1-infected *dcls* and *agos* deletion mutants. (**A**) Colony morphologies of virus-free and FgGMTV1-infected *dcls* and *agos* deletion mutants on PDA with or without 0.05% SDS and 0.1% H_2O_2 after incubation for 3 or 5 days. (**B**) Percentage of mycelial growth inhibition was calculated for 0.05% SDS and 0.1% H_2O_2 treatment. (**C**) Virulence of virus-free and FgGMTV1-infected *dcls* and

agos deletion mutants were evaluated on wheat heads. Wheat heads inoculated with the indicated strains were examined for head blight symptoms at 14 dpi. (**D**) Number of diseased spikelets per wheat head was counted at 14 dpi (n = 15). (**E**) Conidiation in 5-day-old CMC cultures of virus-free and FgGMTV1-infected mutant strains. For **B**, **D**, and **E**, values are represented as the mean ± SD (n = 3 independent experiments). Different letters indicate the statistically significant differences according to one-way ANOVA followed by Dunnett's post-hoc test ($p < 0.05$).

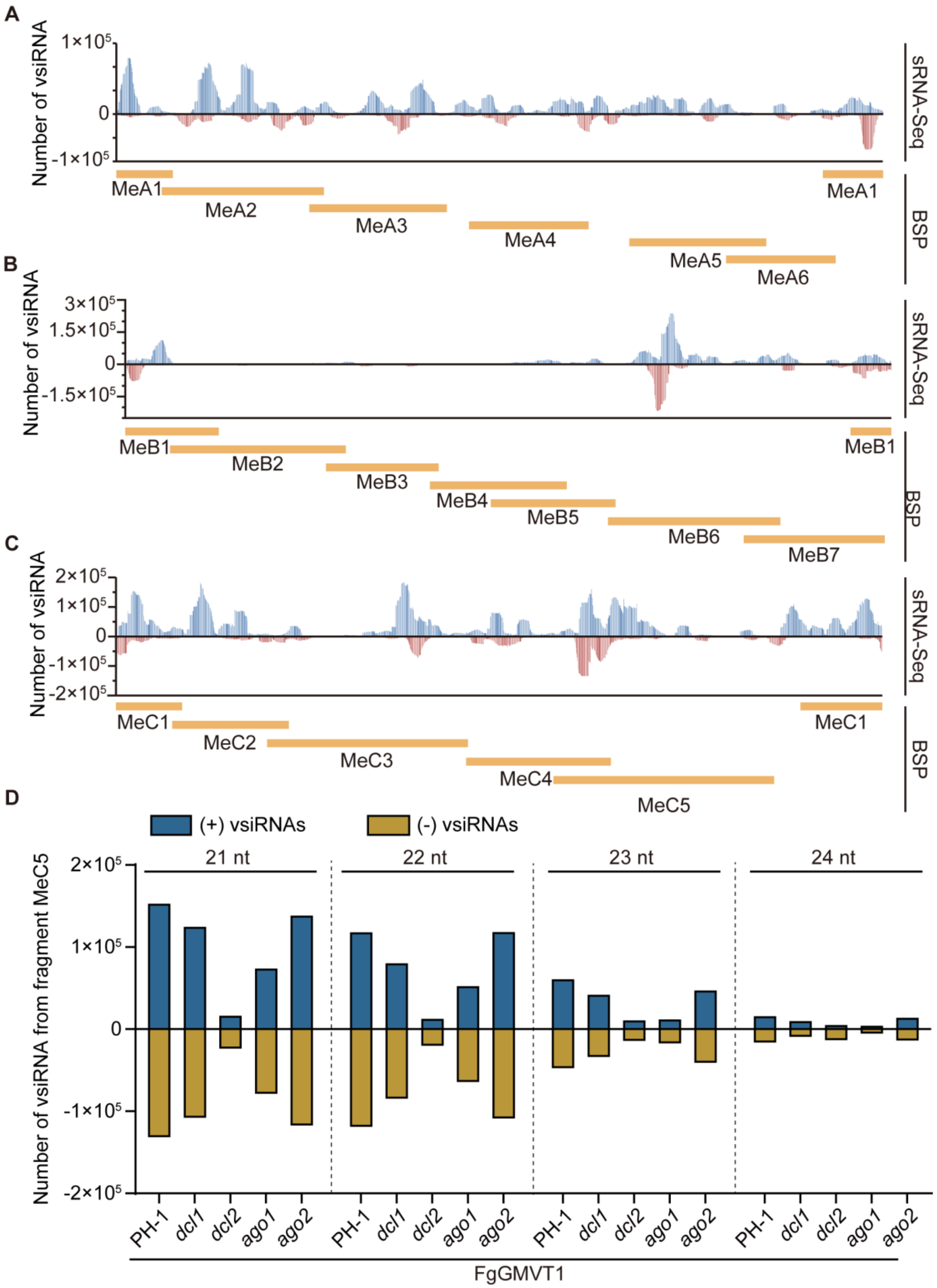

Figure S8 DNA methylation is closely related to sRNAs on the FgGMTV1 genome. (**A-C)** The distribution of vsiRNAs and DNA methylation hot spots derived DNA-A (**A**),

DNA-B (**B**), and DNA-C (**C**) of FgGMTV1. Yellow fragments represent DNA methylation analysis fragments by bisulfite sequencing PCR (BSP). vsiRNAs were obtained by small RNA sequencing (RNA-Seq) (50). The abundance of reads matching the positive viral genomic strand is represented by the blue bar, whereas the abundance of reads mapping to the complementary (negative) strand is represented by the red bars. (**D**) Normalized read number of 21–24 nt vsiRNAs derived from fragment MeC5 in sRNA libraries of the FgGMTV1-infected mutant strains. "(−)" and "(+)" indicate the vsiRNAs derived from the complementary (negative) and positive viral genomic strands, respectively.

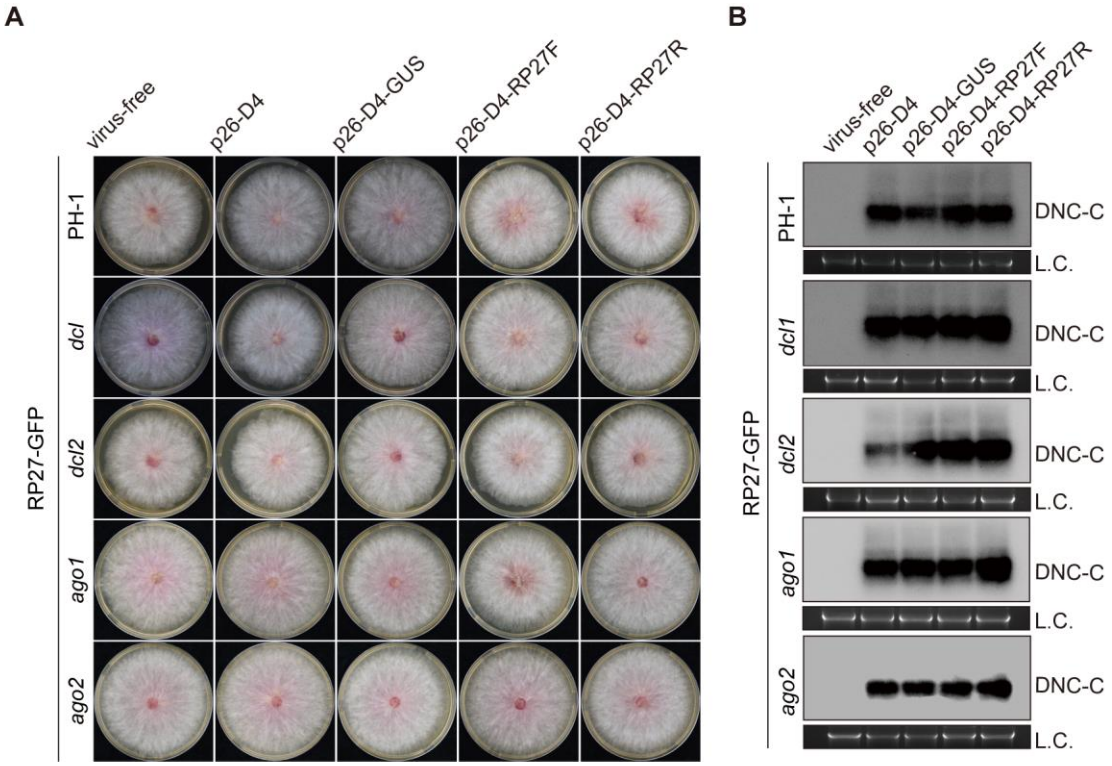

Figure S9 Colony morphologies and virus genome detection of the VIGS vector transfectants. **(A**) Colony morphologies of the virus-free, p26-D4, p26-D4-GUS, and p26-D4-RP27F/R-infected strains. (**B**) Southern blots of genomic DNA isolated from 4-day-old PDA cultures in the dark were hybridized with probes C that are specific for DNA-C of FgGMTV1. L.C., loading control to show equal amounts of genomic DNA in each lane.

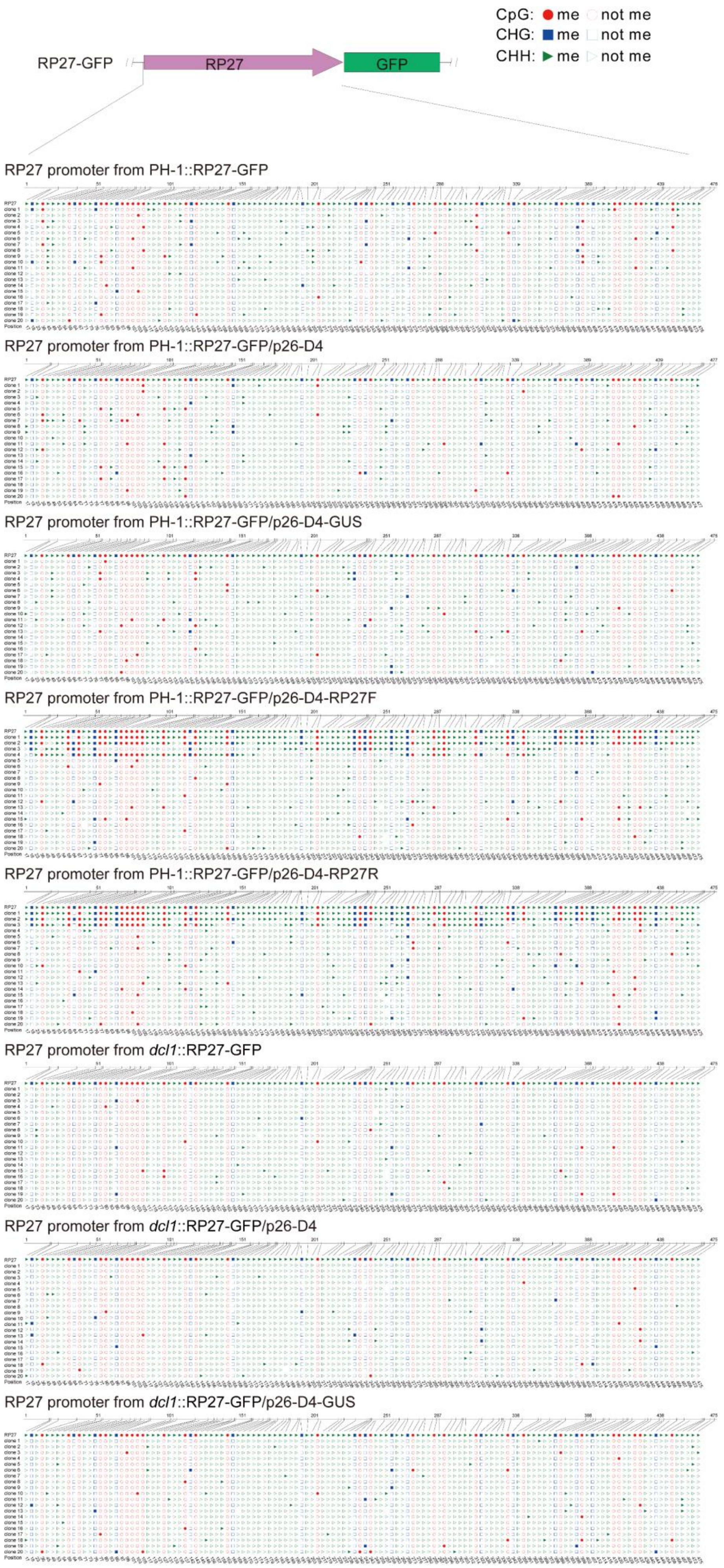
CpG: me not me
CHG: me not me
CHH: me not me
RP27-GFP
RP27
GFP
RP27 promoter from PH-1::RP27-GFP
RP27 promoter from PH-1::RP27-GFP/p26-D4
RP27 promoter from PH-1::RP27-GFP/p26-D4-GUS
RP27 promoter from PH-1::RP27-GFP/p26-D4-RP27F
RP27 promoter from PH-1::RP27-GFP/p26-D4-RP27R
RP27 promoter from *dcl1*::RP27-GFP
RP27 promoter from *dcl1*::RP27-GFP/p26-D4
RP27 promoter from *dcl1*::RP27-GFP/p26-D4-GUS

RP27 promoter from *dcl1*::RP27-GFP/p26-D4-RP27F

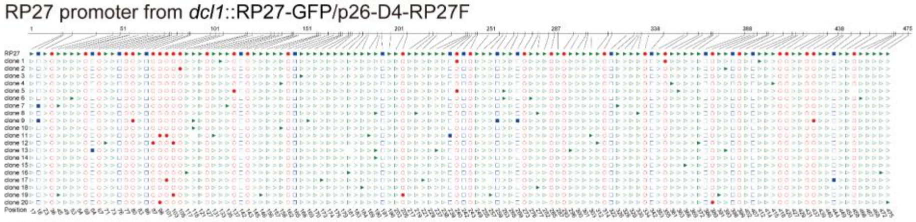

RP27 promoter from *dcl1*::RP27-GFP/p26-D4-RP27R

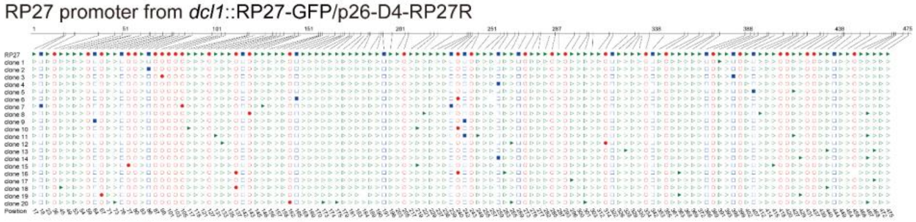

RP27 promoter from *dcl2*::RP27-GFP

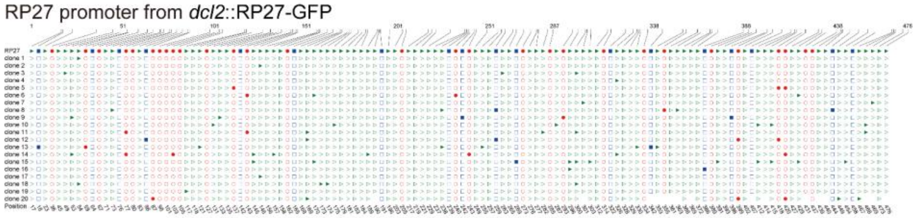

RP27 promoter from *dcl2*::RP27-GFP/p26-D4

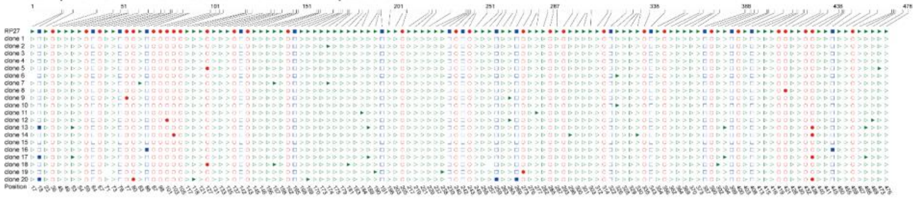

RP27 promoter from *dcl2*::RP27-GFP/p26-D4-GUS

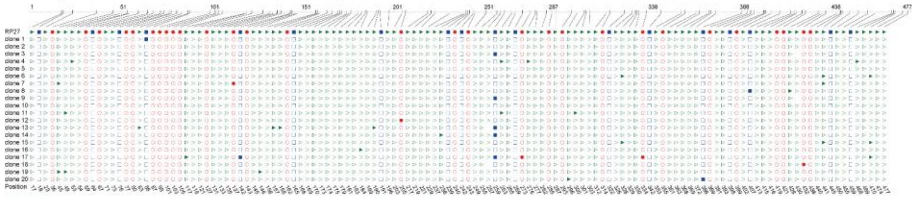

RP27 promoter from *dcl2*::RP27-GFP/p26-D4-RP27F

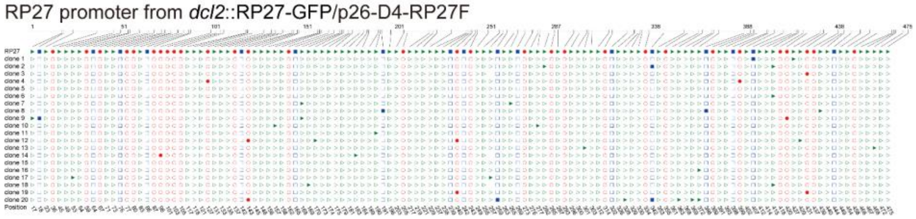

RP27 promoter from *dcl2*::RP27-GFP/p26-D4-RP27R

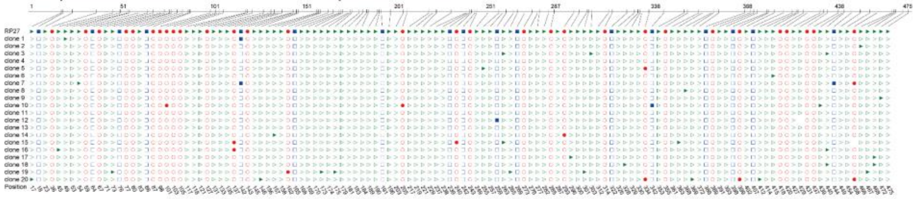

RP27 promoter from *ago1*:RP27-GFP

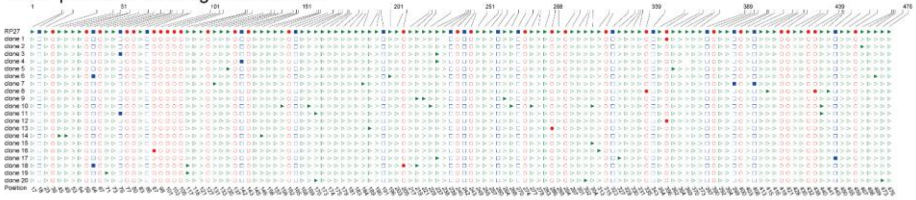

RP27 promoter from *ago1*::RP27-GFP/p26-D4

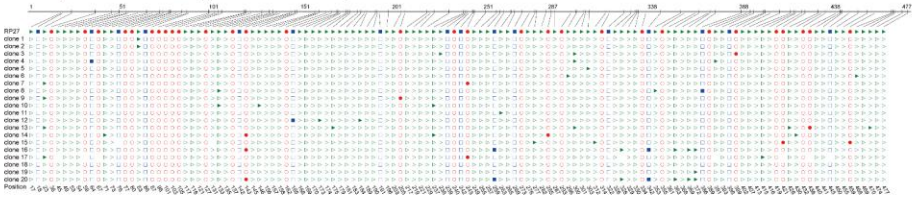

RP27 promoter from *ago1*::RP27-GFP/p26-D4-GUS

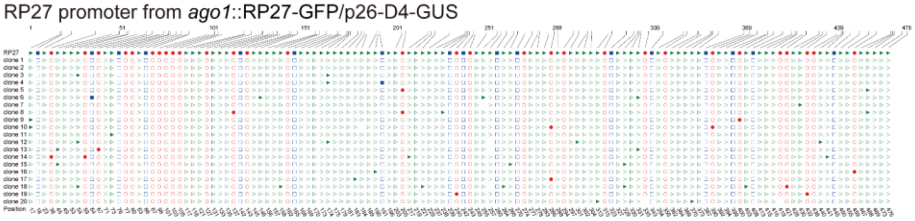

RP27 promoter from *ago1*::RP27-GFP/p26-D4-RP27F

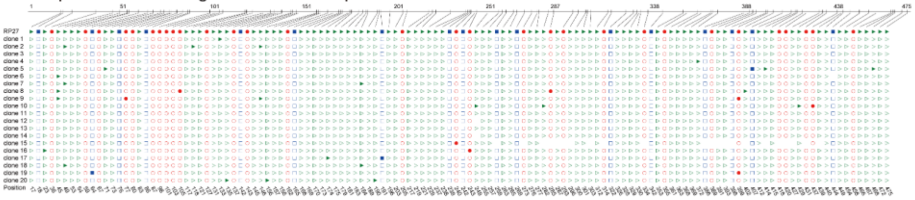

RP27 promoter from *ago1*::RP27-GFP/p26-D4-RP27R

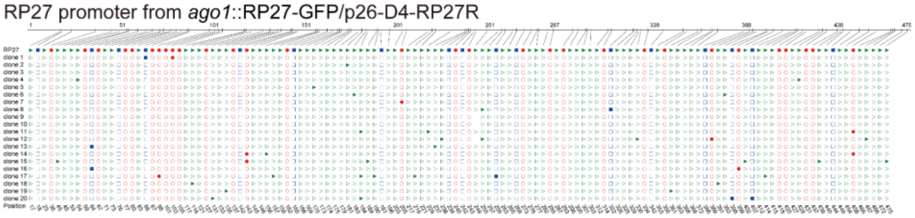

RP27 promoter from *ago2*::RP27-GFP

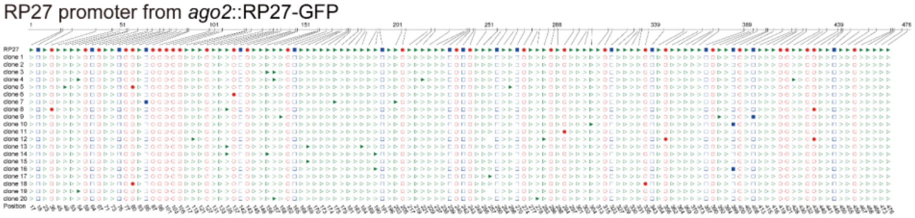

RP27 promoter from *ago2*::RP27-GFP/p26-D4

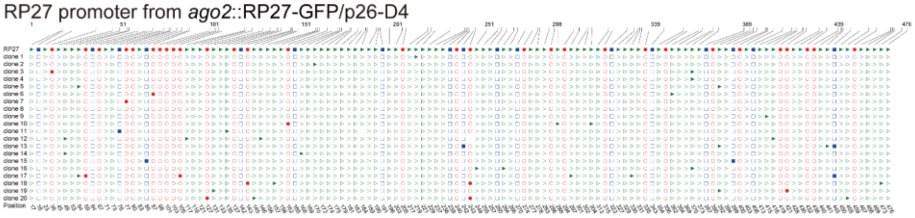

RP27 promoter from *ago2*::RP27-GFP/p26-D4-GUS

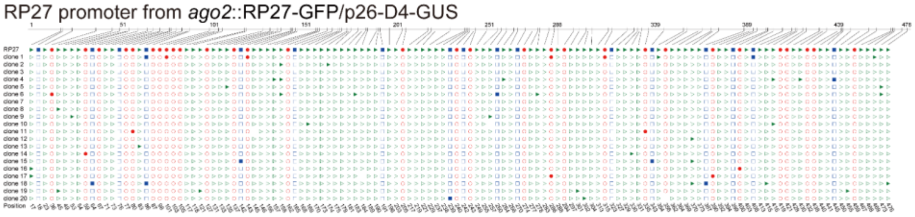

RP27 promoter from *ago2*::RP27-GFP/p26-D4-RP27F

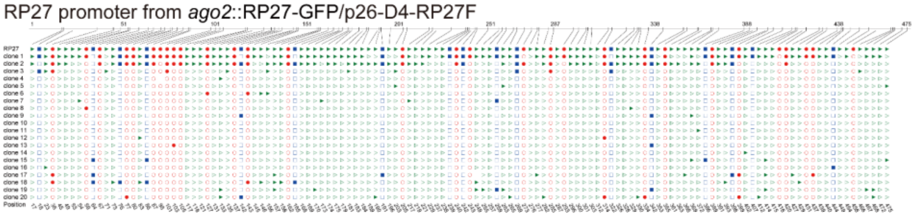

RP27 promoter from *ago2*::RP27-GFP/p26-D4-RP27R

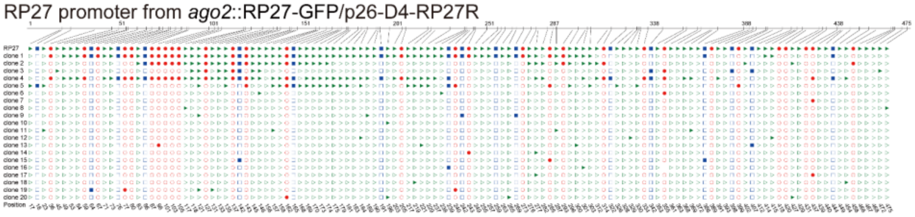

Figure S10 Bisulfite sequencing analysis of methylated cytosine at the RP27 promoter in p26-D4-RP27F/R-infected strains. Cytosine methylation profiles of the RP27 promoter in virus-free, p26-D4, p26-D4-GUS, and p26-D4-RP27F/R-infected strains on PDA after incubation for 4 days. Bisulfite sequencing data of individual clones were submitted to the CyMATE program (http://www.cymate.org) to analyze the methylated cytosines. Circles indicate the cytosine residues and are color coded according to the sequence context (red for CG, blue for CHG, and green for CHH). Solid circles indicate the methylated cytosines. Each line indicates the sequence of an individual clone.

Supplementary Tables

Table S1 All *F. graminearum* strains used in this study.

strians	Brief	Reference
PH-1	Virus-free wild-type	(42)
dim2	The *dim2* deletion mutant of PH-1 retained wild-type levels of mycelial growth, responses to SDS/H_2O_2, conidia production, virulence, and ascospore production.	This study
dim2/DIM2	The complemented strain of the *dim2* deletion mutant retained wild-type levels of mycelial growth, responses to SDS/H_2O_2, conidia production, virulence, and ascospore production.	This study
dcl1	The *dcl1* deletion mutant of PH-1 retained wild-type levels of mycelial growth, responses to SDS, conidia production, and virulence but elevated H_2O_2 sensitivity and impaired ascospore production.	(46)
dcl2	The *dcl2* deletion mutant of PH-1 retained wild-type levels of mycelial growth, responses to SDS, conidia production, virulence, and ascospore production but elevated H_2O_2 sensitivity.	(46)
dcl1/2	The *dcl1* and *dcl2* double-deletion mutant of PH-1 retained wild-type levels of mycelial growth, responses to SDS, conidia production, and virulence but elevated H_2O_2 sensitivity and impaired ascospore production.	(46)
ago1	The *ago1* deletion mutant of PH-1 retained wild-type levels of mycelial growth, responses to SDS, conidia production, virulence, and ascospore production but elevated H_2O_2 sensitivity.	This study
ago2	The *ago2* deletion mutant of PH-1 retained wild-type levels of mycelial growth, responses to SDS, conidia production, and virulence but elevated H_2O_2 sensitivity and impaired ascospore production.	This study
ago1/2	The *ago1* and *ago2* double-deletion mutant of PH-1 retained wild-type levels of mycelial growth, responses to SDS, conidia production, and virulence but elevated H_2O_2 sensitivity and impaired ascospore production.	This study
PH-1/FgGMTV1	The FgGMTV1-infected PH-1 strain showed normal mycelial growth, responses to SDS/H_2O_2, conidia production, and virulence but reduced ascospore production, compared to virus-free PH-1 strain.	This study
dim2/FgGMTV1	The FgGMTV1-infected *dim2* strain showed normal mycelial growth, conidia production, virulence, and ascospore production but elevated viral accumulation and SDS/H_2O_2 hypersensitivity, compared to PH-1/FgGMTV1 strain.	This study
dim2/DIM2/FgGMTV1	The FgGMTV1-infected *dim2*/*DIM2* strain showed normal mycelial growth, responses to SDS/H_2O_2, conidia production, virulence, ascospore production, and viral accumulation, compared to PH-1/FgGMTV1 strain.	This study
dcl1/FgGMTV1	The FgGMTV1-infected *dcl1* showed normal mycelial growth, conidia production, and virulence but reduced ascospore production, elevated viral accumulation, and	This study

	SDS/H_2O_2 hypersensitivity, compared to PH-1/FgGMTV1 strain.	
dcl2/FgGMTV1	The FgGMTV1-infected *dcl2* strain showed normal mycelial growth, conidia production, virulence, and ascospore production but elevated viral accumulation and SDS/H_2O_2 hypersensitivity, compared to PH-1/FgGMTV1 strain.	This study
dcl1/2/FgGMTV1	The FgGMTV1-infected *dcl1/2* strain showed normal conidia production but reduced mycelial growth, attenuated virulence, suppressed ascospore production, elevated viral accumulation, and SDS/H_2O_2 hypersensitivity, compared to PH-1/FgGMTV1 strain.	This study
ago1/FgGMTV1	The FgGMTV1-infected *ago1* strain showed normal mycelial growth and conidia production but reduced virulence, suppressed ascospore production, elevated viral accumulation, and SDS/H_2O_2 hypersensitivity, compared to PH-1/FgGMTV1 strain.	This study
ago2/FgGMTV1	The FgGMTV1-infected *ago2* strain showed normal mycelial growth, responses to SDS/H_2O_2, conidia production, virulence, and ascospore production but elevated viral accumulation, compared to PH-1/FgGMTV1 strain.	This study
ago1/2/FgGMTV1	The FgGMTV1-infected *ago1/2* strain showed normal conidia production but reduced mycelial growth, attenuated virulence, suppressed ascospore production, elevated viral accumulation, and SDS/H_2O_2 hypersensitivity, compared to PH-1/FgGMTV1 strain.	This study
PH-1::RP27-GFP	RP27-GFP transformant of PH-1	This study
dim2::RP27-GFP	RP27-GFP transformant of *dim2*	This study
PH-1::pRep(500)-GFP	pRep(500)-GFP transformant of PH-1	This study
PH-1::pRep(500)-GFP/FgGMTV1	FgGMTV1-infected PH-1::pRep(500)-GFP strain	This study
dim2::pRep(500)-GFP	pRep(500)-GFP transformant of *dim2*	This study
dim2::pRep(500)-GFP/FgGMTV1	FgGMTV1-infected *dim2*::pRep(500)-GFP strain	This study
dcl1::RP27-GFP	RP27-GFP transformant of *dcl1*	This study
dcl2::RP27-GFP	RP27-GFP transformant of *dcl2*	This study
ago1::RP27-GFP	RP27-GFP transformant of *ago1*	This study
ago2::RP27-GFP	RP27-GFP transformant of *ago2*	This study
PH-1::RP27-GFP/p26-D4	Transfectant of the VIGS vector p26-D4, strain PH-1::RP27-GFP as a recipient	This study
PH-1::RP27-GFP/p26-D4-GUS	Transfectant of the VIGS vector p26-D4-GUS, strain PH-1::RP27-GFP as a recipient	This study
PH-1::RP27-GFP/p26-D4-RP27F	Transfectant of the VIGS vector p26-D4-RP27F, strain PH-1::RP27-GFP as a recipient	This study

PH-1::RP27-GFP/p26-D4-RP27R	Transfectant of the VIGS vector p26-D4-RP27R, strain PH-1::RP27-GFP as a recipient	This study
dcl1::RP27-GFP/p26-D4	Transfectant of the VIGS vector p26-D4, strain *dcl1*::RP27-GFP as a recipient	This study
dcl1::RP27-GFP/p26-D4-GUS	Transfectant of the VIGS vector p26-D4-GUS, strain *dcl1*::RP27-GFP as a recipient	This study
dcl1::RP27-GFP/p26-D4-RP27F	Transfectant of the VIGS vector p26-D4-RP27F, strain *dcl1*::RP27-GFP as a recipient	This study
dcl1::RP27-GFP/p26-D4-RP27R	Transfectant of the VIGS vector p26-D4-RP27R, strain *dcl1*::RP27-GFP as a recipient	This study
dcl2::RP27-GFP/p26-D4	Transfectant of the VIGS vector p26-D4, strain *dcl2*::RP27-GFP as a recipient	This study
dcl2::RP27-GFP/p26-D4-GUS	Transfectant of the VIGS vector p26-D4-GUS, strain *dcl2*::RP27-GFP as a recipient	This study
dcl2::RP27-GFP/p26-D4-RP27F	Transfectant of the VIGS vector p26-D4-RP27F, strain *dcl2*::RP27-GFP as a recipient	This study
dcl2::RP27-GFP/p26-D4-RP27R	Transfectant of the VIGS vector p26-D4-RP27R, strain *dcl2*::RP27-GFP as a recipient	This study
ago1::RP27-GFP/p26-D4	Transfectant of the VIGS vector p26-D4, strain *ago1*::RP27-GFP as a recipient	This study
ago1::RP27-GFP/p26-D4-GUS	Transfectant of the VIGS vector p26-D4-GUS, strain *ago1*::RP27-GFP as a recipient	This study
ago1::RP27-GFP/p26-D4-RP27F	Transfectant of the VIGS vector p26-D4-RP27F, strain *ago1*::RP27-GFP as a recipient	This study
ago1::RP27-GFP/p26-D4-RP27R	Transfectant of the VIGS vector p26-D4-RP27R, strain *ago1*::RP27-GFP as a recipient	This study
ago2::RP27-GFP/p26-D4	Transfectant of the VIGS vector p26-D4, strain *ago2*::RP27-GFP as a recipient	This study
ago2::RP27-GFP/p26-D4-GUS	Transfectant of the VIGS vector p26-D4-GUS, strain *ago2*::RP27-GFP as a recipient	This study
ago2::RP27-GFP/p26-D4-RP27F	Transfectant of the VIGS vector p26-D4-RP27F, strain *ago2*::RP27-GFP as a recipient	This study
ago2::RP27-GFP/p26-D4-RP27R	Transfectant of the VIGS vector p26-D4-RP27R, strain *ago2*::RP27-GFP as a recipient	This study

Table S2 All primers used in this study.

Primer	Sequence (5’-3’)	Relevant Characteristics
MeA1-F	GGTTGAGGTGTTTTTGAGTTATATGAT	Used for bisulfite sequencing of DNA-A MeA1 fragment
MeA1-R	TATAAAAATCACTACCCCTCTCTATACCTT	
MeA2-F	AGGGGTAGTGATTTTTATAATAATGG	Used for bisulfite sequencing of DNA-A MeA2 fragment
MeA2-R	CCTACTTCCCAAACATATTCATACTCT	
MeA3-F	AGTATGAATATGTTTGGGAAGTAGG	Used for bisulfite sequencing of DNA-A MeA3 fragment
MeA3-R	CATCAAATCATTTACTAAATAAAAATTC	
MeA4-F	TGGTAGTTAAGTGTAAAAATTTTTTTG	Used for bisulfite sequencing of DNA-A MeA4 fragment
MeA4-R	TTTCTATAATATTCACCCCAACATC	
MeA5-F	TTATTTTTGTGTTGTTTTTTTGAGATG	Used for bisulfite sequencing of DNA-A MeA5 fragment
MeA5-R	AAAATCCCTCCCCCATAATAATAC	
MeA6-F	TGGGAATAGTTTTATTTTTTGGTAATT	Used for bisulfite sequencing of DNA-A MeA6 fragment
MeA6-R	TATAACTCAAAAACACCTCAACCAC	
MeB1-F	GGGTAGGATTAGTTAGTAAATTTTTAATTA	Used for bisulfite sequencing of DNA-B MeB1 fragment
MeB1-R	TTCTTCTTTATAAAAACCATTTTTATC	
MeB2-F	TGTTAGTTTGTTTTTGGATTTAAAAA	Used for bisulfite sequencing of DNA-B MeB2 fragment
MeB2-R	ACTCCCATATTAACTAAAATTTCACC	
MeB3-F	TTTGTTGTGGGTGAAATTTTAGTTA	Used for bisulfite sequencing of DNA-B MeB3 fragment
MeB3-R	CACAACCCTCAAATAAATCCCTATA	
MeB4-F	TTATTTGAGGGTTGTGATATTTATT	Used for bisulfite sequencing of DNA-B MeB4 fragment
MeB4-R	ACTACACCTAAAATCTTCCTCTACC	
MeB5-F	GGATGAGATATTGGATTAGTTTTTGT	Used for bisulfite sequencing of DNA-B MeB5 fragment
MeB5-R	AACTCCAAACCATCCTACCTATC	
MeB6-F	GATGGTTTGGAGTTTGTGTTTAGT	Used for bisulfite sequencing of DNA-B MeB6 fragment
MeB6-R	AACCCTTACTTCAACTCAATTATCAC	
MeB7-F	TTGAGTTGGTGATAGTTTTATTTTT	

MeB7-R	CTCTTTCTTAAATTTCTTAACCTCC	Used for bisulfite sequencing of DNA-B MeB7 fragment
MeC1-F	AGTGGTTGGGGTATTTATGGTTAA	Used for bisulfite sequencing of DNA-C MeC1 fragment
MeC1-R	TCATATCACCACATTTCCACATATC	
MeC2-F	GAAATGTGGTGATATGAAGAATTGT	Used for bisulfite sequencing of DNA-C MeC2 fragment
MeC2-R	TAATCCTAATTAAATAAACATACCAAATCA	
MeC3-F	TTGGTTGATGATTTGGTATGTTTATT	Used for bisulfite sequencing of DNA-C MeC3 fragment
MeC3-R	AACCCCTTCTAACAACAACTTTTCT	
MeC4-F	GGTTAGATGTGGTTGAGTAAAGATTT	Used for bisulfite sequencing of DNA-C MeC4 fragment
MeC4-R	AAACATCAATCCAAAAAAATTCATC	
MeC5-F	TGGAGGGGAATGTTTTTAGTAT	Used for bisulfite sequencing of DNA-C MeC5 fragment
MeC5-R	AAATCCACATTTTATTTTACCTCAC	
qPCR-EF1α-F	GCTTACTGCCTCCACCAACT	Used for TR-qPCR for analysis of *EF-1α* gene expression
qPCR-EF1α-R	CGTTCCAATACCGCCAAT	
qPCR-DIM2-F	AGACACCTAATGCTCAGCCG	Used for TR-qPCR for analysis of *dim2* gene expression
qPCR-DIM2-R	GCAATGAAGATGGCAATGC	
qPCR-DCL1-F	CAGCCATCGTTGAAGTGAC	Used for TR-qPCR for analysis of *dcl1* gene expression
qPCR-DCL1-R	CAAGGTTGACAGTATCCCAGTC	
qPCR-DCL2-F	GAAGGTTTGGTTAGCGACC	Used for TR-qPCR for analysis of *dcl2* gene expression
qPCR-DCL2-R	GGCGAACTCGTAAGCATAG	
qPCR-AGO1-F	GCTCACCAAGAAGGACAAGAG	Used for TR-qPCR for analysis of *ago1* gene expression
qPCR-AGO1-R	ATCTCGTCAACTCGGGCTAC	
qPCR-AGO2-F	CTTCAAGCAACTTCGCAAG	Used for TR-qPCR for analysis of *ago2* gene expression
qPCR-AGO2-R	GTAGTCATAGACGCTGATGTCC	
HY-F	GGCTTGGCTGGAGCTAGTGGAGGTCAA	Used for PCR amplification of the 5' fragment of *HPH*
HY-R	GTATTGACCGATTCCTTGCGGTCCGAA	
YG-F	GATGTAGGAGGGCGTGGATATGTCCT	Used for PCR amplification of the 3' fragment of *HPH*
YG-R	AACCCGCGGTCGGCATCTACTCTATTC	

HYG-ID-F	TTCCTCCCTTTATTTCAGATTCAA	Used for identification of *HPH*
HYG-ID-R	ATGTTGGCGACCTCGTATTGG	
HYG-U-R	GCTGATCTGACCAGTTGC	Used for identification of upstream fragment of *HPH*
HYG-D-F	GTCGATGCGACGCAATCGT	Used for identification of downstream fragment of *HPH*
GE-F	GAGGTTGCGATTTCTCTGCCGTATCTG	Used for PCR amplification of the 5’ fragment of Geneticin resistance gene
GE-R	CAGTCGATGAATCCAGAAAAGCG	
EN-F	GGAAGGGACTGGCTGCTATTGG	Used for PCR amplification of the 3’ fragment of Geneticin resistance gene
EN-R	GCCAGCAGTAGACACTTGGAATCTAAAC	
GEN-ID-F	GAATGGTCAAATCAAACTGCTAGATAT	Used for identification of Geneticin resistance gene
GEN-ID-R	TGTTGGGTTTGAGCTAGGTGGG	
GEN-D-F	TCGGCTATGACTGGGCACAACA	Used for identification of upstream fragment of Geneticin resistance gene
GEN-U-R	GAGCGGCGATACCGTAAAGCAC	Used for identification of downstream fragment of Geneticin resistance gene
DIM2-U-F	TGTGTACGACAGCCCAGTGGAAGGAG	Used for PCR amplification of the upstream fragment of *dim2* for the construction of *dim2* strain
DIM2-U-R	TTGACCTCCACTAGCTCCAGCCAAGCCATCGGACGCCTCGTCCTATGTTAA	
DIM2-D-F	GAATAGAGTAGATGCCGACCGCGGGTTTTTTTGTACAATTGATGTCAGGC	Used for PCR amplification of the downstream fragment of *dim2* for the construction of *dim2* strain
DIM2-D-R	CCTATGTCTTCAAGGGACCTCCTGGA	
DIM2-ID-F	AGAGAGATGGTGCGGATTC	Used for identification of *dim2* gene in *dim2* and *dim2/DIM2* strains
DIM2-ID-R	CAGGACCCGAAGTATTAGCC	
DIM2-ID-F1	GACGACCTTGATGCCTTGCGCGA	Used for identification of upstream fragment of *dim2* gene in *dim2* and *dim2/DIM2* strains
DIM2-ID-R1	CGAATCATACCAGTACATGGTACAA	Used for identification of downstream fragment of *dim2* gene in *dim2* and *dim2/DIM2* strains
DIM2-COM-U-F	TGTGTACGACAGCCCAGTGGAAGGAG	Used for PCR amplification of the upstream fragment of *dim2* for the construction of *dim2/DIM2* strain
DIM2-COM-U-R	CAGATACGGCAGAGAAATCGCAACCTCCTACTCATTTGAAACGCTGTGCCTC	
DIM2-COM-D-F	GTTTAGATTCCAAGTGTCTACTGCTGGCATTTTTGTACAATTGATGTCAGGC	Used for PCR amplification of the downstream fragment of *dim2* for the construction of *dim2/DIM2* strain
DIM2-COM-D-R	CCTATGTCTTCAAGGGACCTCCTGGA	

AGO1-U-F	CTGCCTAAAGTGAAGGAATTG	Used for PCR amplification of the upstream fragment of *ago1* for the construction of *ago1* strain
AGO1-U-R	TTGACCTCCACTAGCTCCAGCCAAGCCACACTA ACATCAACTTCGGCAGT	
AGO1-D-F	GAATAGAGTAGATGCCGACCGCGGGTTAGAAA GATTAATCGTATAAGAAGCAGA	Used for PCR amplification of the downstream fragment of *ago1* for the construction of *ago1* strain
AGO1-D-R	CCGTCCACCACCACCAATAATGGC	
AGO1-ID-F	GACATTTATCAACACTCTTTCTTG	Used for identification of *ago1* gene in *ago1* strain
AGO1-ID-R	GAACTCGGTCGCAAGAAGCAGGCT	
AGO1-ID-F1	GTAACTGACGATATGACCTGGA	Used for identification of upstream fragment of *ago1* gene in *ago1* strain
AGO1-ID-R1	AATGATCGTAAAACTCACTATCC	Used for identification of downstream fragment of *ago1* gene in *ago1* strain
AGO2-U-F	AATGATCGTAAAACTCACTATCC	Used for PCR amplification of the upstream fragment of *ago2* for the construction of *ago2* strain
AGO2-U-R	TTGACCTCCACTAGCTCCAGCCAAGCCGGTGA CAGATGAGTCTGAAGAAGTT	
AGO2-D-F	GAATAGAGTAGATGCCGACCGCGGGTTCCAGA ACAATGGCGTTGATGCAAG	Used for PCR amplification of the downstream fragment of *ago2* for the construction of *ago2* strain
AGO2-D-R	CGGTATAATCGATAACGTCGCTGT	
AGO2-ID-F	GGCACTGAACTTCATTGATCACCTT	Used for identification of *ago2* gene in *ago2* and *ago1/2* strains
AGO2-ID-R	GGCCAAACCTGACAGTAGGGGGAG	
AGO2-ID-F1	GGTGCTATGTTTGTCAGTTATGTC	Used for identification of upstream fragment of *ago2* gene in *ago2* and *ago1/2* strains
AGO2-ID-R1	GCATCTGTCTGTATTTACCCTACA	Used for identification of downstream fragment of *ago2* gene in *ago2* and *ago1/2* strains
AGO1/2-U-F	AATGATCGTAAAACTCACTATCC	Used for PCR amplification of the upstream fragment of *ago2* for the construction of *ago1/2* strain
AGO1/2-U-R	CAGATACGGCAGAGAAATCGCAACCTCGGTGA CAGATGAGTCTGAAGAAGTT	
AGO1/2-D-F	GTTTAGATTCCAAGTGTCTACTGCTGGCCCAGA ACAATGGCGTTGATGCAAG	Used for PCR amplification of the downstream fragment of *ago2* for the construction of *ago1/2* strain
AGO1/2-D-R	CGGTATAATCGATAACGTCGCTGT	
Probe A-F	TGGGAAGTAGGCGTGATT	Used for Southern blot detection of DNA-A genome
Probe A-R	CACACCAACCATCCTTGA	
Probe B-F	GGCAATCCGCAAACACAT	Used for Southern blot detection of DNA-B genome
Probe B-R	CTCCGTCTTCAACAACGCA	

Probe C-F	GTATGTCCACCCAATCAGG	Used for Southern blot detection of DNA-C genome
Probe C-R	CCCTTCTGGCAACAACTT	
qPCR-DNA-A-F	TGGGAAGTAGGCGTGATT	Used for quantitative PCR for analysis of DNA-A accumulation
qPCR-DNA-A-R	AGCACATACGAATCTCCAC	
qPCR-DNA-B-F	GGCAATCCGCAAACACAT	Used for quantitative PCR for analysis of DNA-B accumulation
qPCR-DNA-B-R	GACATATCACGAACCGCCAA	
qPCR-DNA-C-F	GCCACAAGCACCTCTTTAGA	Used for quantitative PCR for analysis of DNA-C accumulation
qPCR-DNA-C-R	TCGCCGGTTACAATACAAG	
pRep(500)-GFP-F	AGGGAACAAAAGCTGGGTACCACACATCATAC CGACCCATAAGTC	Used for amplification of promoter of Rep
pRep(500)-GFP-R	CATTCTAGAACTAGTGGATCCTGTGGCTGAAGA GTGGCTTTGCGGA	
qPCR-GFP-F	CGTAAACGGCCACAAGTTCA	Used for quantitative PCR for analysis of GFP expression
qPCR-GFP-R	CTTCATGTGGTCGGGGTAGC	
VIGS-GUS-F	AGCACTAACACGTGTACCGGTGCTGTGCCAGG CAGTTTTAAC	Used for amplification of sense 150 bp GUS fragment
VIGS-GUS-R	TATGTCCACCCAATCATGCATGCCGTAATGAGT GACCGCATCG	
VIGS-RP27F-F	AGCACTAACACGTGTACCGGTATAAATGTAGGT ATTACCTGT	Used for amplification of sense 150 bp RP27F fragment
VIGS-RP27F-R	TATGTCCACCCAATCATGCATCTTTGGCGGCCC CGTGTGTG	
VIGS-RP27R-F	AGCACTAACACGTGTACCGGTCTTTGGCGGCCC CGTGTGTG	Used for amplification of antisense 150 bp RP27R fragment
VIGS-RP27R-R	TATGTCCACCCAATCATGCATATAAATGTAGGTA TTACCTGT	
